# A genetic sum score of risk alleles associated with body mass index interacts with socioeconomic position in the Heinz Nixdorf Recall Study

**DOI:** 10.1371/journal.pone.0221252

**Published:** 2019-08-23

**Authors:** Mirjam Frank, Nico Dragano, Marina Arendt, Andreas J. Forstner, Markus M. Nöthen, Susanne Moebus, Raimund Erbel, Karl-Heinz Jöckel, Börge Schmidt

**Affiliations:** 1 Institute for Medical Informatics, Biometry and Epidemiology, University of Duisburg-Essen, Essen, Germany; 2 Institute of Medical Sociology, Centre for Health and Society, University Hospital Düsseldorf, Düsseldorf, Germany; 3 Institute of Human Genetics, University of Bonn, School of Medicine & University Hospital Bonn, Bonn, Germany; 4 Centre for Human Genetics, University of Marburg, Marburg, Germany; 5 Department of Biomedicine, University of Basel, Basel, Switzerland; 6 Department of Psychiatry (UPK), University of Basel, Basel, Switzerland; University of Hong Kong, CHINA

## Abstract

Body mass index (BMI) is influenced by genetic, behavioral and environmental factors, while interactions between genetic and socioeconomic factors have been suggested. Aim of the study was to investigate whether socioeconomic position (SEP) interacts with a BMI-related genetic sum score (GRS_BMI_) to affect BMI in a population-based cohort. SEP-related health behaviors and a GRS associated with educational attainment (GRS_Edu_) were included in the analysis to explore potential interactions underlying the GRS_BMI_xSEP effect. Baseline information on SEP indicators (education, income), BMI, smoking, physical activity, alcohol consumption and genetic risk factors were available for 4,493 participants of the Heinz Nixdorf Recall Study. Interaction analysis was based on linear regression as well as on stratified analyses. In SEP-stratified analyses, the highest genetic effects were observed in the lowest educational group with a 0.24 kg/m^2^ higher BMI (95%CI: 0.16; 0.31) and in the lowest income quartile with a 0.14 kg/m^2^ higher BMI (95%CI: 0.09; 0.18) per additional risk allele. Indication for a GRS_BMI_xSEP interaction was observed for education (ß_GRSbmixeducation_ = -0.02 [95%CI:-0.03; -0.01]) and income (ß_GRSbmixincome_ = -0.05 [95%CI: -0.08; -0.02]). When adjusting for interactions with the GRS_Edu_ and SEP-related health behaviors, effect size estimates of the GRS_BMI_xSEP interaction remained virtually unchanged. Results gave indication for an interaction of BMI-related genetic risk factors with SEP indicators, showing substantially stronger genetic effects in low SEP groups. This supports the hypothesis that expression of genetic risks is higher in socioeconomically disadvantaged environments. No indication was observed that the GRS_BMI_xSEP interaction was affected by other SEP-related factors included in the analysis.

## Introduction

Obesity is a complex condition caused by various genetic, environmental and behavioral factors [1,2]. Research has shown that indicators of socioeconomic position (SEP), such as income and education, are also strongly related to obesity, indicating the highest prevalence in groups of low SEP, especially in western countries [3–5]. Genome-wide association studies (GWAS) have detected numerous single nucleotide polymorphisms (SNPs) robustly associated with body mass index (BMI), indicating a polygenetic contribution to obesity risk. The effects of single genetic variants are modest to small, as well as the variance explained by the combined effect of all genetic loci detected [1,6]. It has been suggested that interactions between genes and the environment (GxE) may partly account for the unexplained variance of BMI, meaning that for some genetic variants, the genetic effect depends on environmental exposures [7–9]. In the study of GxE interaction, SEP indicators can serve as proxy markers for a wide range of socially unequally distributed environmental, psychosocial and, in particular, behavioral health risks (e.g., low physical activity, unhealthy diet, smoking) [10] which are usually more prevalent among lower socioeconomic groups [11–13]. Twin studies have suggested an interaction of genetic factors with education on BMI [14] and with education and income on overall physical health [15,16]. However, previous studies on GxSEP interactions have rarely included underlying risk factors potentially responsible for the interactions detected.

The aims of the present study were to investigate whether (1) the SEP indicators income and education interact with a genetic sum score of BMI-related risk alleles (GRS_BMI_) to affect BMI in a population-based cohort and (2) whether these GRS_BMI_xSEP interactions might be mediated by physical activity, smoking or alcohol consumption as SEP associated health behaviors. Further, (3) a GRS associated with educational attainment (GRS_Edu_) was included to investigate potential gene by-gene interaction effects (GRS_BMI_xGRS_Edu_).

## Materials and methods

### Study population

Baseline data of the Heinz Nixdorf Recall Study, a population-based prospective cohort, was used. The rationale of the study has been described in detail elsewhere [17]. In brief, 4,814 participants aged 45–74 years were randomly selected from mandatory registries of residence of the cities Bochum, Essen and Mülheim/Ruhr within the largest metropolitan region in the western part of Germany. The baseline response proportion (December 2000 to June 2003) was 55.8% [18]. The study was approved by the ethics committees of the University Hospital Essen and included extended quality management procedures and certification according to DIN ISO 9001:2000. Informed consent was obtained from all participants.

### Data collection

At study baseline, BMI (kg/m^2^) was calculated based on standardized weight (in underclothes) and height measures. Information on educational attainment, household income, physical activity and smoking was assessed in standardized computer-assisted face-to-face interviews. Education was defined by combining school and vocational training as total years of formal education according to the International Standard Classification of Education [19]. Years of education was used as a continuous variable or categorized into four groups with ≤10 years, 11–13 years, 14–17 years and ≥18 years of education. The lowest educational group is equivalent to a minimum compulsory school attendance and no additional vocational degree and the highest educational group is comparable to a vocational training including additional qualification or a university degree. Income was measured as the monthly household equivalent income calculated by dividing the participants’ household net income by a weighting factor for each household member [20]. Income was included as a continuous variable or divided into four groups, using sex-specific quartiles. In order to take account for their different mechanisms in causing health inequalities, both SEP indicators were analyzed separately [21,22]. Smoking status was dichotomized for analyses as current smoker (smoking cigarettes during the past year) versus former and never smoker. Physical activity was defined as no regular engagement in physical exercise versus exercising one and more times per week. Amount of alcohol intake is given in gram per week and was estimated from information on the total number of alcoholic drinks by type of drink (beer, wine, sparkling wine, and spirits) usually consumed in a week and was included as a continuous variable into analyses.

#### Genetic data

Lymphocyte DNA was isolated from EDTA anti-coagulated venous blood using the Chemagic Magnetic Separation Module I (Chemagen, Baesweiler, Germany). Genotyping was performed using different Illumina microarrays (Metabochip, Omni1-Quad, Omni1S, OmniExpressv1.0, HumanCoreExomev1.0, HumanCoreExomev1.1; Illumina, San Diego, USA) according to the manufacturer’s protocols. Quality control was applied prior to imputation, separately for each chip and was first performed on subject level including sex-, ethnicity- and relatedness-checks, excluding subjects with missing genotype data >5%. Further, SNPs with a minor allele frequency (MAF) <1%, a missing genotype frequency >5% or a deviation from Hardy–Weinberg Equilibrium (HWE) (p<10^−5^) were excluded. Imputation was carried out using IMPUTE v.2.3.1 [23] with reference data from 1000 Genomes Phase 1, release March 2012, for the Metabochip and 1000 Genomes Phase 3, release October 2014, for all other microarray data.

Using the meta-analyses of genome-wide association studies (GWAS) by Locke et al., 2015 [6], 97 SNPs or suitable proxy SNPs (r^2^>0.90), representing independent genetic loci robustly associated with BMI, were identified (S1 Table and S2 Table). In addition, 74 education-associated SNPs, or their proxy SNPs (r^2^> 0.94), were selected from GWAS meta-analysis by Okbay et al., 2016 [24] (S3 Table). For two loci associated with educational attainment, neither the original SNP nor their proxy SNP was available within the study population. Unweighted genetic risk scores associated with BMI (GRS_BMI_) and with educational attainment (GRS_Edu_) were calculated by aggregating the total number of risk alleles for each individual across the selected SNPs (for detailed information see S2 Fig and S3 Fig). The GRS_BMI_ was additionally weighted by the effect size estimates of each SNP reported by Locke et al. [6]. As the results using the weighted score did not differ to those resulting from the unweighted GRS_BMI_, only the latter were reported.

### Statistical analyses

Overall, 4,493 participants with non-missing information on BMI, gender, age and genetic variants were included in the analyses (S1 Fig). As participants had missing information on education (n = 13) and income (n = 279), smoking status (n = 76), physical activity (n = 71) and alcohol consumption (n = 109), analysis populations differed in the respective analyses. Participants with missing information on income or education did not differ substantially in age, BMI or in GRS_BMI_ compared to the analysis population. As most of the SNPs associated with educational attainment were found on the GWAS microarrays, which were available for n = 4,147 participants only, analyses including the GRS_Edu_ were carried out in this sub-sample.

Linear regression models were fitted, adjusted for sex and age, to estimate effects and 95% confidence intervals (95% CIs) on BMI for education and income (model 1), the GRS_BMI_ (model 2) and SEP-related health behaviors smoking, physical activity and alcohol consumption (models 3–5). To assess GRS_BMI_xSEP interactions, the GRS_BMI_ and SEP main effects as well as GRS_BMI_xSEP interaction terms were included (model 6). The SEP indicators education and income were used separately as continuous variables. Interaction analysis was also repeated for each BMI-associated SNP and SEP indicator. The genetic effect on BMI was calculated stratified by education groups and income quartiles; the effect of SEP indicators on BMI was stratified by tertiles of the GRS_BMI_.

All possible combinations of GRS_BMI_ tertiles and SEP groups were entered as dummy variables into regression models to calculate the joint effects of the GRS_BMI_ and SEP indicators on BMI, separately for income and education, using the group with the highest SEP and lowest GRS tertile as single reference [25].

To analyze whether the GRS_BMI_xSEP interaction may be affected by underlying interactions between GRS_BMI_ and SEP-related health behaviors, smoking (S), physical activity (PA) or alcohol (A) main effects and the respective GRS_BMI_xS/PA/A interaction terms, in addition to an SEPxPA/S/A interaction term, were included in model 6, separately for each health behavior and SEP indicator (models 7–9) [26]. Moreover, a model was fitted including GRS_BMI_, GRS_Edu_, SEP main effects, GRS_BMI_xGRS_Edu_ and GRS_BMI_xEducation interaction terms to investigate whether there is indication for overall gene-by-gene interaction (model 10). For all analyses the statistical computing software R v3.1.1 [27] was used. For single SNP analyses Plink v1.07 software package for Windows was used [28].

## Results

The mean BMI (± standard deviation) in the study population was 27.9 ± 4.6 (Table 1). Men showed a higher BMI than women (28.2 ± 4.0 vs. 27.7 ± 5.2). Sex differences were also observed in the distribution of the two SEP indicators with women reporting lower formal education and a lower median income. One fourth of the study population was current smoker, while almost half of the study population stated to be physically inactive. The median alcohol consumption was 13.9 gram per week.

**Table 1:** Characteristics of study population, stratified by sex.

	All (n = 4493)	Men (n = 2251)	Women (n = 2242)
**Age (years)** [n_miss_ = 0]*	59.6 ± 7.8	60.0 ± 7.8	59.6 ± 7.8
**Number of BMI risk allele (GRS**_**BMI**_**)** [n_miss_ = 0]*	91.3 ± 6.2	91.0 ± 6.3	91.3 ±6.2
**Education associated alleles (GRS**_**Edu**_**)** [n_miss_ = 346]*	72.4 ± 5.6	72.5 ± 5.6	72.3 ± 5.6
**Body mass index (kg/m**^**2**^**)** [n_miss_ = 0]	27.9 ± 4.6	28.2 ± 4.0	27.7 ± 5.2
**Education (years of training)** [n_miss_ = 11] ⱡ			
≤10	512 (11.4%)	116 (5.2%)	396 (17.7%)
11–13	2486 (55.5%)	1065 (47.5%)	1421 (63.4%)
14–17	1005 (22.4%)	758 (33.8%)	247 (11.0%)
≥18	479 (10.7%)	303 (13.5%)	176 (7.9%)
**Income (€/month)** [n_miss_ = 279]†	1449.0 (1108.0–1875.0)	1520.0 (1107.8–2072.8)	1313.8 (937.4–1874.7)
**Smoking status** [n_miss_ = 5]ⱡ			
Never	1835 (41.5%)	615 (27.8%)	1220 (55.2%)
Former + Current	2582 (58.5%)	1593 (72.2%)	989 (44.8%)
**No Physical Activity** [n_miss_ = 0]ⱡ	2162 (48.9%)	1143 (51.7%)	1019 (46.1%)
**Alcohol consumption (g/week)** [n_miss_ = 109]†	13.9 (0.0–63.7)	46.3 (6.9–119.8)	0.9 (0.0–15.6)

*mean ± standard deviation (SD).

†median (first quartile- third quartile).

or ⱡproportion (%)[n_miss_ = number of participants with missing values].

Socioeconomic inequalities in BMI were found in the study population with a lower BMI observed with higher household income and years of education (Table 2, model 1). On average, a 0.10 kg/m^2^ higher BMI was seen per additional risk allele (Table 2, model 2). Adjusted for SEP, reporting no physical activity resulted in a higher BMI, while current smoking and higher alcohol consumption showed a negative effect on BMI (Table 2, model 3–5).

**Table 2:** Sex- and age- adjusted effects and corresponding 95% confidence intervals (95% CI) on body mass index (BMI) in linear regression models including main effects of income (per 100€/month) and education (per year) as indicators of socioeconomic position (SEP), a BMI-associated genetic risk score (GRS_BMI_), and factors of SEP-related health behavior (no physical inactivity, current smoking, alcohol consumption [per 100g/week]).

**Model 1** BMI ~ Income + age + sex			
	**n**	**β (95%-CI)**	***p***
Intercept	4214	25.54 (24.38; 26.71)	<2.0*10^−16^
Age		0.06 (0.04; 0.08)	2.5*10^−11^
Sex		-0.60 (-0.87; -0.32)	2.8*10^−5^
Income		-0.59 (-0.79; -0.40)	4.4*10^−9^
BMI ~ Education + age + sex			
Intercept	4482	28.68 (27.18; 30.18)	<2.0*10^−16^
Age		0.05 (0.04; 0.07)	1.1*10^−10^
Sex		-0.91 (-1.19; -0.63)	3.4*10^−10^
Education		-0.25 (-0.31; -0.19)	<2.0*10^−16^
**Model 2** BMI ~ GRS_BMI_ + age +sex			
Intercept	4493	15.30 (13.07; 17.53)	< 2.0*10^−16^
Age		0.07 (0.05; 0.09)	3.1*10^−15^
Sex		-0.53 (-0.80; -0.26)	0.0001
GRS_BMI_		0.10 (0.07; 0.12)	< 2*10^−16^
**Model 3** BMI ~ Education + Physical activity + age + sex			
Intercept	4482	27.85 (26.33; 29.37)	<2.0*10^−16^
Education		-0.22 (-0.28; -0.16)	7.8*10^−13^
No Physical activity		0.80 (0.53; 1.07)	8.7*10^−9^
Age		0.05 (0.04; 0.07)	1.7*10^−9^
Sex		-0.82 (-1.10; -0.53)	1.8*10^−8^
**Model 4** BMI ~ Education + Smoking + age + sex			
Intercept	4482	30.02 (28.47; 31.57)	<2.0*10^−16^
Education		-0.27 (-0.33; -0.21)	<2.0*10^−16^
Smoking		-1.02 (-1.34; -0.69)	8.0*10^−10^
Age		0.04 (0.02; 0.06)	7.1*10^−6^
Sex		-0.98 (-1.26; -0.70)	1.2*10^−11^
**Model 5** BMI ~ Education + Alcohol consumption + age + sex			
Intercept	4376	28.55 (26.92; 30.19)	< 2.0*10^−16^
Education		-0.25 (-0.32; -0.19)	5.8*10^−14^
Alcohol consumption		-0.08 (-0.23; 0.07)	0.31
Age		0.06 (0.04; 0.08)	2.8*10^−9^
Sex		-1.07 (-1.39; -0.74)	1.5*10^−10^

**Fig 1:**
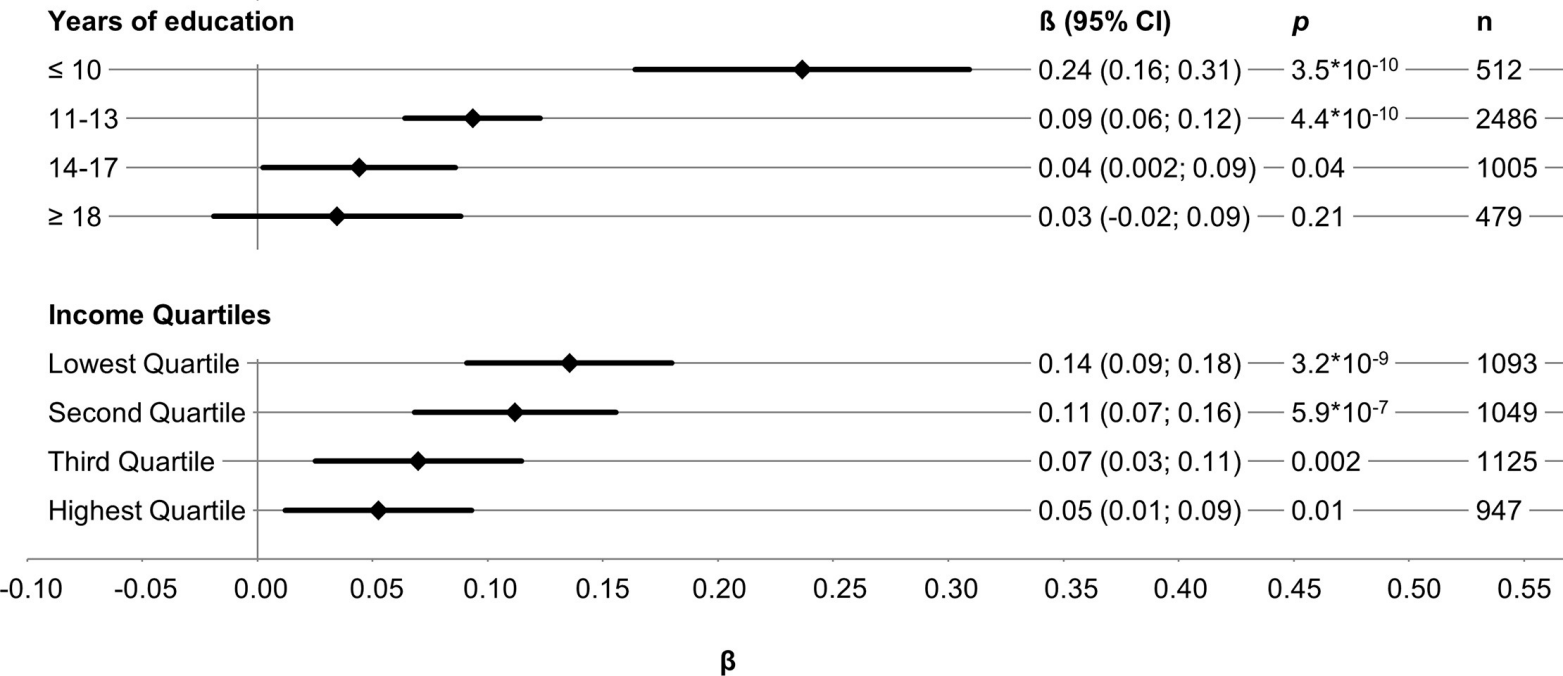
Sex- and age-adjusted effects and corresponding 95% confidence interval (95% CI) of the genetic effect on body mass index (BMI), stratified by education groups (years) and income quartiles in linear regression models.

In stratified analyses, the genetic effect on BMI was strongest in groups with low SEP (Fig 1).

Participants with ≤10 years of education showed the strongest genetic effect which almost disappeared in the highest educational group. Similar results were observed for income, although the trend across income quartiles was less pronounced. Results for the association of SEP indicators with BMI stratified by GRS_BMI_ tertiles revealed the strongest effect sizes estimates in the highest GRS_BMI_ stratum (S4 Table). In the analysis of joint effects with a single reference group, beta estimates showed a clear trend within and between groups: with increasing years of education and decreasing number of risk alleles, the weaker the effect size estimates. Compared to the reference group with the highest education and lowest GRS_BMI_, participants with a high GRS_BMI_ and less than 10 years of education showed on average a 4.54 units higher BMI. Less clear patterns were observed for the joint effects of income and GRS_BMI_ on BMI (Table 3).

**Table 3:** Sex- and age-adjusted effects and corresponding 95% confidence intervals (95% CI) on body mass index (BMI) in linear regression models of the joint effects of tertiles of a BMI-associated genetic risk score (GRS_BMI_) and socioeconomic position indicators, calculated separately for income quartiles and education categories, with the group of having a low genetic risk score and the highest socioeconomic position as reference.

**Income Quartiles**			
	**n**	**ß (95% CI)**	***p***
Lower Quartile			
High GRS_BMI_	175	0.19 (-0.51; 0.89)	0.59
Middle GRS_BMI_	170	-0.70 (-1.38; -0.02)	0.05
Low GRS_BMI_	167	-0.65 (-1.33; 0.03)	0.06
2nd Quartile			
High GRS_BMI_	827	0.98 (0.33; 1.62)	<0.01
Middle GRS_BMI_	849	0.31 (-0.35; 0.97)	0.35
Low GRS_BMI_	810	-0.37 (-1.04; 0.29)	0.27
3rd Quartile			
High GRS_BMI_	333	1.24 (0.57; 1.91)	<0.01
Middle GRS_BMI_	329	-0.08 (-0.75; 0.58)	0.81
Low GRS_BMI_	343	-0.30 (-0.96; 0.37)	0.38
Highest Quartile			
High GRS_BMI_	135	1.77 (1.10; 2.44)	2.0*10^−7^
Middle GRS_BMI_	172	0.69 (0.03; 1.34)	0.04
Low GRS_BMI_	172	Ref. -	-
**Education Groups**			
	**n**	**ß (95% CI)**	***p***
≤ 10 years			
High GRS_BMI_	295	4.54 (3.57; 5.51)	<2.0*10^−16^
Middle GRS_BMI_	320	2.72 (1.74; 3.69)	4.6*10^−8^
Low GRS_BMI_	332	1.62 (0.64; 2.59)	<0.01
11–13 years			
High GRS_BMI_	404	2.68 (1.93; 3.43)	2.2*10^−12^
Middle GRS_BMI_	366	1.80 (1.06; 2.55)	2.2*10^−6^
Low GRS_BMI_	355	1.18 (0.43; 1.93)	<0.01
14–17 years			
High GRS_BMI_	340	1.99 (1.15; 2.82)	3.2*10^−6^
Middle GRS_BMI_	355	1.10 (0.26; 1.93)	0.01
Low GRS_BMI_	354	1.39 (0.56; 2.22)	<0.01
≥ 18 years			
High GRS_BMI_	351	0.76 (-0.26; 1.78)	0.14
Middle GRS_BMI_	381	0.02 (-0.93; 0.98)	0.96
Low GRS_BMI_	361	Ref.-	-

For the GRS_BMI_ by income interaction term (ß = -0.05 [95% CI: -0.08; -0.02] per 1000€/month increase and additional risk allele) as well as for the GRS_BMI_ by education interaction term (ß = -0.02 [95% CI: -0.03; -0.01] per year of education and additional risk allele) negative effect size estimates were observed (Table 4, model 6).

**Table 4:** Sex- and age- adjusted effects and corresponding 95% confidence intervals (95% CI) on body mass index (BMI) in linear regression models including main effects and interaction terms of a BMI-associated genetic risk score (GRS_BMI_), indicators of socioeconomic position (SEP; years of education and 1000€ income/month), SEP-related health behaviors (no physical activity [PA], current smoking [S], alcohol consumption [A; per 100g/week]) and a genetic risk score related to educational attainment (GRS_Edu_).

	Model 6—BMI ~ SEP + age + sex + GRS_BMI_ + GRS_BMI_ *SEP		Model 7—BMI ~ SEP + age + sex + GRS_BMI_ + PA + GRS_BMI_ *SEP + GRS_BMI_ *PA + SEP*PA		Model 8—BMI ~ SEP + age + sex + GRS_BMI_ + S + GRS_BMI_ *SEP + GRS_BMI_ *S + SEP*S		Model 9—BMI ~ SEP + age + sex + GRS_BMI_ + A + GRS_BMI_ *SEP + GRS_BMI_ *A + SEP*A		Model 10—BMI~ GRS_BMI_ + GRS_Edu_ + GRS_BMI_*GRS_Edu_ + sex+ age	
**Education**	**β (95%-CI)**	***p***	**β (95%-CI)**	***p***	**β (95%-CI)**	***p***	**β (95%-CI)**	***p***	**β (95%-CI)**	***p***
n	4482		4482		4482		4376		4147	
Intercept	-4.93 (-16.35; 6.49)	0.4	-0.22 (-12.19; 11.76)	0.97	-1.69 (-13.21; 9.84)	0.77	-3.09 (-14.70; 8.51)	0.60	0.47 (-25.68; 26.63)	0.97
Age	0.05 (0.04; 0.07)	5.9*10^−10^	0.05 (0.03; 0.07)	3.0*10^−8^	0.04 (0.02; 0.06)	3.8*10^−5^	0.06 (0.04; 0.07)	3.8*10^−10^	0.07 (0.05; 0.08)	1.3*10^−13^
Sex	-0.91 (-1.19; -0.63)	1.8*10^−10^	-0.81 (-1.09; -0.52)	5.9*10^−9^	-1.00 (-1.28; -0.71)	6.5*10^−12^	-0.96 (-1.26; -0.66)	3.6*10^−10^	-0.53 (-0.80; -0.25)	1.9*10^−4^
Education	1.52 (0.73; 2.32)	<0.001	1.32 (0.51; 2.13)	0.001	1.44 (0.64; 2.24)	4.2*10^−4^	1.34 (0.52; 2.16)	0.001	-	-
GRS_BMI_	0.37 (0.24; 0.49)	7.9*10^−9^	0.31 (0.18; 0.44)	2.7*10^−6^	0.36 (0.23; 0.48)	2.5*10^−8^	0.35 (0.23, 0.48)	4.5*10^−8^	0.26 (-0.03; 0.55)	0.08
GRS_Edu_	-	-	-	-	-	-	-	-	0.20 (-0.16; 0.56)	0.27
PA	-	-	-4.16 (-8.46; 0.13)	0.06	-	-	-	-	-	-
S	-	-	-	-	-7.18 (-12.25; -2.12)	0.01	-	-	-	-
A	-	-	-	-	-	-	0.14 (-1.77; 2.04)	0.89	-	-
GRS_BMI_ x PA	-	-	0.05 (0.002; 0.09)	0.04	-	-	-	-	-	-
GRS_BMI_ x S	-	-	-	-	0.03 (-0.02; 0.08)	0.26	-	-	-	-
GRS_BMI_ x A	-	-	-	-	-	-	-0.01 (-0.03; 0.01)	0.20	-	-
Education x PA	-	-	0.06 (-0.06; 0.17)	0.32	-	-	-	-	-	-
Education x S	-	-	-	-	0.25 (0.11; 0.39)	4.0*10^−4^	-	-	-	-
Education x A	-	-	-	-	-	-	0.06 (0.01; 0.12)	0.02	-	-
GRS_BMI_ x Education	-0.02 (-0.03; -0.01)	1.27*10^−5^	-0.02 (-0.03; -0.01)	1.5*10^−4^	-0.02 (-0.03; -0.01)	1.6*10^−5^	-0.02 (-0.03; 0.01)	8.9*10^−5^	-	-
GRS_BMI_ x GRS_Edu_	-	-	-	-	-	-	-	-	-0.002 (-0.006; 0.002)	0.27
**Income**	**β (95%-CI)**	***p***	**β (95%-CI)**	***p***	**β (95%-CI)**	***p***	**β (95%-CI)**	***p***		
n	4214		4218		4218		4128			
Intercept	10.15 (5.17; 15.10)	6.5*10^−5^	13.90 (8.30; 19.50)	1.2*10^−6^	12.84 (7.67; 18.01)	1.1*10^−6^	9.96 (4.92; 15.00)	0.0001		
Age	0.06 (0.04; 0.08)	4.5*10^−12^	0.06 (0.04; 0.08)	1.8*10^−10^	0.05 (0.03; 0.06)	5.6*10^−7^	0.06 (0.05; 0.08)	2.5*10^−5^		
Sex	-0.59 (-0.86; -0.31)	3.0*10^−5^	-0.50 (-0.78; -0.22)	0.00042	-0.62 (-0.90; -0.34)	1.3*10^−5^	-0.64 (-0.93; -0.34)	2.3*10^−12^		
Income	3.08 (-1.42; 7.58)	<0.01	2.84 (0.05; 5.63)	0.05	3.22 (0.46; 5.99)	0.02	3.08 (0.29; 5.87)	0.03		
GRS_BMI_	0.17 (0.11; 0.22)	5.8*10^−10^	0.13 (0.07; 0.19)	3.0*10^−5^	0.15 (0.10; 0.21)	3.4*10^−8^	0.17 (0.12; 0.23)	3.6*10^−10^		
GRS_Edu_	-	-	-	-	-	-	-	-		
PA	-	-	-5.24 (-9.33; -1.15)	0.01	-	-	-	-		
S	-	-	-	-	-5.37 (-10.20; -0.54)	0.03	-	-		
A	-	-	-	-	-	-	1.17 (-0.60; 2.93)	0.20		
GRS_BMI_ x PA	-	-	0.06 (0.01; 0.10)	0.01	-	-	-	-		
GRS_BMI_ x S	-	-	-	-	0.03 (-0.02; 0.09)	0.19	-	-		
GRS_BMI_ x A	-	-	-	-	-	-	-0.02 (-0.04; 0.002)	0.07		
Income x PA	-	-	0.59 (0.20; 0.98)	0.003	-	-	-	-		
Income x S	-	-	-	-	0.76 (0.32; 1.21)	0.001	-	-		
Income x A	-	-	-	-	-	-	0.23 (0.07; 0.40)	0.01		
GRS_BMI_ x Income	-0.05 (-0.08; -0.02)	0.002	-0.04 (-0.07; -0.01)	0.01	-0.04 (-0.07; -0.01)	0.004	-0.04 (-0.07; -0.01)	0.01		

After including physical activity, smoking and alcohol consumption, effect size estimates of the GRS_BMI_xSEP interaction virtually did not change (Table 4, models 7–9). Moreover, an indication for GRS_BMI_xPA interaction was found in regression models adjusted for income and for education (Table 4, model 7). However, no changes in interaction effects were observed after excluding income main effects and respective interaction effects from models 7–9. The GRS_Edu_ showed an association with education (ß = 0.03 [95%-CI: 0.02, 0.05] per additional risk allele), but no indication for GRS_BMI_xGRS_Edu_ interaction (Table 4, model 10).

Interaction effect size estimates between individual BMI-associated SNPs and SEP indicators were close to each other in the range of -0.20 to 0.15 for education and -0.33 to 0.38 for income (S1 Table and S2 Table). Interaction effect size estimates for some single SNPs pointed in the opposite direction than the respective interaction effect of the GRS. Effect sizes of the interaction effect between rs1558902 of the *FTO* gene and both SEP indicators were rather small compared to other SNPs of the GRS_BMI_.

## Discussion

The aim of the present study was to investigate whether a sum score of genetic variants associated with BMI interacts with indicators of SEP in a population-based cohort and to explore SEP-related health behaviors and genetic factors which may explain such GRS_BMI_xSEP interactions. Results gave indication for a negative interaction between the GRS_BMI_ and the SEP indicators income and education. This was supported by results of stratified analysis in which the strongest genetic effects on BMI were seen in groups of low SEP. Joint effects of all possible combinations of GRS_BMI_ tertiles and education groups showed the strongest effect on BMI for participants with highest genetic risk within the lowest education group. Effect size estimates for the GRS_BMI_xSEP interactions remained virtually unchanged after including SEP-related health behaviors (i.e., smoking, physical activity and alcohol consumption) and education-related genetic factors into regression analysis, suggesting that these factors do not explain the observed GRS_BMI_xSEP interaction. Independent of the SEP effect, a GRS_BMI_ by physical activity interaction was observed.

The direction of the GRS by SEP interaction effect was consistent with results of previous studies: Liu et al. have investigated interaction between a GRS of BMI-associated risk alleles and education, using data of the Health and Retirement Study. While the GRSxEducation interaction on BMI revealed non-significant results, the direction of interaction effect size estimates has been consistent to the present results [29]. Likewise, another study that has examined interactions between BMI-associated GRSs and SEP reported smaller genetic effects within groups of higher educational attainment in two different study samples, albeit interaction effects were again statistically non-significant [30]. Within study samples of the UK Biobank, GRS_BMI_xSEP interaction has been observed using the area-related Townsend deprivation index (TDI) as SEP indicator [31,32]. Additionally, Tyrrell et al. have assessed interactions of GRS_BMI_ with job class and education, measured by the ISCED, which, however, yield non-significant results. Rask-Andersen et al. have evaluated interactions between GRS_BMI_ and 131 environmental factors and reported an GRS_BMI_xIncome interaction [31], which is comparable to the present results.

Even though education and income are correlated SEP indicators, each of them represents certain aspects of SEP related to different health behaviors and risks. Regarding the impact of SEP on BMI, several causal pathways have been described ranging from an improved knowledge about promoting health skills, increased social support in groups of higher education, to lower levels of stress and stress reactivity and better access to recreational facilities and healthy food with higher income [14,15,33]. Moreover, higher SEP leads to greater control over important determinants of health due to a better ability to engage in healthier behaviors. Via these pathways, SEP may also have an effect on the genetic susceptibility to higher BMI, including the possibility of altered gene expression by epigenetic modifications [34,35]. However, including SEP-related health behaviors physical activity, smoking and alcohol consumption in the present analysis no substantial changes in the GRS_BMI_xSEP interaction effect size estimates were revealed, indicating that other SEP-related risk factors potentially mediate the found GRS_BMI_xSEP interaction. In a recent study by Komulainen et al. within a Finnish study sample, a GRS_BMI_xEducation interaction has also been reported [36]. Similar to the present results, the observed interaction effect size estimates attenuated only slightly after further adjusting for main effects of physical activity and their respective interaction effects.

While in the present study no indication for a GRS_BMI_xS or GRS_BMI_xA interaction was observed, some indication for positive GRS_BMI_xPA interaction was obtained, supporting results of previous studies [31,37,38].

The present study adds further knowledge to the results of previous studies with the investigation of gene by gene interactions potentially underlying the observed GRS_BMI_xSEP interaction. However, the GRS_Edu_ showed no indication for interaction with GRS_BMI_. This suggested that strong GenexGene interactions in the present study population are unlikely, albeit interactions of single BMI- with education-related genetic loci cannot be ruled out. In using a GRS the cumulative genetic risk of a person was being considered and thus, results of interaction analyses have to be interpreted rather global. The power of the study was not sufficient for robustly analysing single SNPxSNP interactions, indicators of SEP or SEP-related health behaviors. Effect size estimates of SNPxSEP interactions, however, were found to be rather similar in magnitude, indicating that no single genetic variant by itself noticeably triggered the observed GRS_BMI_xSEP interaction, which is in line with the results of a previous study [32]. Different to other studies, which have found interactions especially between variants of the fat-mass and obesity-associated *FTO* gene [37,39–41], the variant of the *FTO* gene used in the present study did not belong to the SNPs with strongest interaction effect.

Strengths of the present study include its population-based study sample and the use of two different individual SEP indicators in the analyses. Further, evidence on interaction was not only based on testing GRS_BMI_xSEP interaction terms, but also on stratified analyses and analysis of joint effects. Another advantage of the present study was that several health behaviors and a GRS of genetic variants related to educational attainment were included in the analyses. However, following limitations need to be considered: Next to the sample size and the limited statistical power for single SNP analyses, the age range of study participants did not enable to make statements about interactions between childhood/youth BMI and SEP during childhood/youth. As the detection of BMI-related genetic loci is still ongoing, the genetic information included in the analysis does not represent all genetic susceptibility to higher BMI.

In conclusion, the results of the present study provide indication for an interaction of genetic factors related to BMI with indicators of SEP in a population-based study sample, showing stronger genetic effects in groups of low SEP. This gives support to the hypothesis that SEP influences the expression of genetic susceptibility related to BMI: Higher SEP groups seem to be better enabled to reduce their genetic effect on BMI. However, physical activity, smoking status or alcohol consumption did not seem to explain the observed SEP-related differences. GRS_BMI_xSEP interaction has thus to be explained by other factors underlying the interaction effect. To fully understand the complex relationship of genetic variants and environmental factors on BMI, further research is needed that takes account of other factors, such as dietary patterns or stress levels, which might modify the effect of BMI-associated genetic variants on BMI.

## Supporting information

S1 Table Sex- and age-adjusted effects and corresponding 95% confidence interval (95% CI) ofthe interaction of each body mass index (BMI)—Associated single-nucleotide polymorphism (SNP) with education on BMI. (DOCX)

S2 Table Sex- and age-adjusted effects and corresponding 95% confidence interval (95% CI) of the interaction of each body mass index (BMI)- associated single-nucleotide polymorphism (SNP) with income (per 1000€) on BMI. (DOCX)

S3 Table Overview of 72 loci associated with educational attainment (Okbay et al, 2016) included into genetic risk score (GRS_Edu_). (DOCX)

S4 Table Sex- and age-adjusted effects and corresponding 95% confidence intervals (CI) of the genetic effect on body mass index (BMI) in linear regression models, stratified by tertiles of the BMI-associated genetic risk score (GRS_BMI_), separately for income (per 1000€) and education (per year of education). (DOCX)

S5 Table Sex- and age- adjusted effects and corresponding 95% confidence intervals (95% CI) on body mass index (BMI) in linear regression models including main effects and respective interaction terms of a BMI-associated genetic risk score (GRS_BMI_) and socioeconomic position-related health behaviors (no physical inactivity [PA], current smoking [S], alcohol consumption [PA; per 100g/week]). (DOCX)

S1 Fig Flowchart of participants out of the entire Heinz Nixdorf Recall Study (HNR) cohort included in the analysis. (TIF)

S2 Fig Selection of SNPs from different Illumina microarrays for calculating the BMI-associated genetic risk score (GRS_BMI_) in the HNR study population. Using the meta-analyses of genome-wide association studies by Locke et al., 2015 (6), 97 SNPs representing independent genetic loci robustly associated with BMI were identified. Of these, 95 SNPs (incl. 2 SNPs were proxy SNPs [r^**2**^ > 0.9]) were found in genotyped data of the Metabochip available for all study participants included in the analysis. Two SNPs (incl. 1 SNP was a proxy SNP [r^**2**^ = 1]) were found in imputed and combined data of different Illumina genome-wide chips (Omni1-Quad n = 779, Omni1S n = 1,348, OmniExpressv1.0 n = 457, HumanCoreExome n = 1,747 with partly overlapping information available for 3,874 participants of the study population) with an imputation quality of 0.98 for both SNPs. As for these two SNPs information for 718 individuals was not available in the study population, this information was imputed based on the sample allele frequency according to the PLINK scoring routine (Purcell et al., 2007). The GRS_BMI_ was then calculated for n = 4,493.
(TIF)

S3 Fig Selection of SNPs from different Illumina BeadArrays for calculating the genetic risk score associated with educational attainment (GRS_Edu_) in the HNR study population. Using the meta-analyses of genome-wide association studies by Okbay et al., 2016 (24), 74 independent genetic loci robustly associated with educational attainment were selected. Of these, 12 SNPs were found in data of the Metabochip (incl. proxies for 6 SNPs [r^**2**^ between 0.94 and 1.00] and 3 imputed SNPs with an imputation quality between 0.94 and 0.99). Further, 60 SNPs were found in combined data of different Illumina genome-wide chips (Omni1-Quad n = 779, Omni1S n = 1,348, OmniExpressv1.0 n = 457, HumanCoreExome n = 1,747 with partly overlapping information available for 4,147 participants of the study population). Of these, 12 SNPs were selected from genotyped data and 48 SNPs (incl. 1 proxy SNP with r^**2**^ = 1.0) were imputed with an imputation quality between 0.92 and 1.00. For 2 loci, neither the original SNP nor the proxy SNP was available. Thus, the GRS_Edu_ was calculated including 72 SNPs associated with educational attainment for n = 4,147.
(TIF)
