# Supplementary material for: A genetic sum score of risk alleles associated with body mass index interacts with socioeconomic position in the Heinz Nixdorf Recall Study
Source: PLoS One. 2019 Aug 23;14(8):e0221252. doi: 10.1371/journal.pone.0221252 (PMC6707579; doi:10.1371/journal.pone.0221252)
Supplement: S1 Table — (DOCX) [file pone.0221252.s001.docx]

**S1 Table. Sex- and age-adjusted effects and corresponding 95% confidence interval (95% CI) ofthe interaction of each body mass index (BMI) - associated single-nucleotide polymorphism (SNP) with education on BMI.**

| **CHR** | **Position*** | **SNP** | **Risk Allele** | **TEST** | **BETA** | **95 % CI** | | **P** |
| --- | --- | --- | --- | --- | --- | --- | --- | --- |
|  |  |  |  |  |  | **Lower** | **Upper** |  |
| 1 | 47684677 | rs977747 | G | SNP | -0.8105 | -2.02 | 0.40 | 0.19 |
|  |  |  |  | Age | 0.0527 | 0.04 | 0.07 | 5.11E-09 |
|  |  |  |  | Sex | -0.8810 | -1.17 | -0.60 | 1.70E-09 |
|  |  |  |  | Education | -0.3161 | -0.44 | -0.19 | 3.58E-07 |
|  |  |  |  | SNPxEducation | 0.0503 | -0.03 | 0.14 | 0.25 |
| 1 | 49589847 | rs657452 | G | SNP | -0.1234 | -1.30 | 1.05 | 0.84 |
|  |  |  |  | Age | 0.0513 | 0.03 | 0.07 | 1.48E-08 |
|  |  |  |  | Sex | -0.8761 | -1.16 | -0.59 | 2.56E-09 |
|  |  |  |  | Education | -0.2550 | -0.37 | -0.14 | 2.66E-05 |
|  |  |  |  | SNPxEducation | -0.0002 | -0.08 | 0.08 | 0.996 |
| 1 | 50559820 | rs11583200 | T | SNP | 0.6100 | -0.56 | 1.78 | 0.31 |
|  |  |  |  | Age | 0.0525 | 0.03 | 0.07 | 5.73E-09 |
|  |  |  |  | Sex | -0.8784 | -1.17 | -0.59 | 1.91E-09 |
|  |  |  |  | Education | -0.1951 | -0.31 | -0.08 | 0.001 |
|  |  |  |  | SNPxEducation | -0.0466 | -0.13 | 0.04 | 0.27 |
| 1 | 72751185 | rs3101336 | T | SNP | -1.1610 | -2.36 | 0.04 | 0.06 |
|  |  |  |  | Age | 0.0527 | 0.04 | 0.07 | 5.02E-09 |
|  |  |  |  | Sex | -0.8720 | -1.16 | -0.59 | 2.46E-09 |
|  |  |  |  | Education | -0.3043 | -0.39 | -0.22 | 8.38E-12 |
|  |  |  |  | SNPxEducation | 0.0685 | -0.02 | 0.15 | 0.11 |
| 1 | 75002193 | rs12566985 | A | SNP | -0.8966 | -2.06 | 0.27 | 0.13 |
|  |  |  |  | Age | 0.0529 | 0.04 | 0.07 | 4.33E-09 |
|  |  |  |  | Sex | -0.8794 | -1.17 | -0.59 | 1.90E-09 |
|  |  |  |  | Education | -0.3204 | -0.43 | -0.21 | 2.77E-08 |
|  |  |  |  | SNPxEducation | 0.0572 | -0.02 | 0.14 | 0.17 |
| 1 | 78446761 | rs12401738 | G | SNP | -0.1480 | -1.32 | 1.03 | 0.81 |
|  |  |  |  | Age | 0.0525 | 0.03 | 0.07 | 5.66E-09 |
|  |  |  |  | Sex | -0.8800 | -1.17 | -0.59 | 1.79E-09 |
|  |  |  |  | Education | -0.2576 | -0.38 | -0.13 | 4.13E-05 |
|  |  |  |  | SNPxEducation | 0.0037 | -0.08 | 0.09 | 0.93 |
| 1 | 96924097 | rs11165643 | C | SNP | 0.1890 | -0.96 | 1.33 | 0.75 |
|  |  |  |  | Age | 0.0526 | 0.03 | 0.07 | 5.23E-09 |
|  |  |  |  | Sex | -0.8835 | -1.17 | -0.60 | 1.53E-09 |
|  |  |  |  | Education | -0.2329 | -0.32 | -0.14 | 5.35E-07 |
|  |  |  |  | SNPxEducation | -0.0235 | -0.10 | 0.06 | 0.57 |
| 1 | 110154688 | rs17024393 | T | SNP | -3.3460 | -6.19 | -0.50 | 0.02 |
|  |  |  |  | Age | 0.0522 | 0.03 | 0.07 | 6.68E-09 |
|  |  |  |  | Sex | -0.8830 | -1.17 | -0.60 | 1.52E-09 |
|  |  |  |  | Education | -0.6336 | -1.03 | -0.24 | 0.002 |
|  |  |  |  | SNPxEducation | 0.1983 | 0.00 | 0.40 | 0.054 |
| 1 | 177889480 | rs543874 | A | SNP | -2.2330 | -3.72 | -0.75 | 0.003 |
|  |  |  |  | Age | 0.0526 | 0.03 | 0.07 | 5.30E-09 |
|  |  |  |  | Sex | -0.8819 | -1.17 | -0.60 | 1.62E-09 |
|  |  |  |  | Education | -0.5068 | -0.69 | -0.32 | 6.04E-08 |
|  |  |  |  | SNPxEducation | 0.1539 | 0.05 | 0.26 | 0.004 |
| 1 | 201784287 | rs2820292 | A | SNP | -0.0124 | -1.16 | 1.13 | 0.98 |
|  |  |  |  | Age | 0.0525 | 0.03 | 0.07 | 5.75E-09 |
|  |  |  |  | Sex | -0.8803 | -1.17 | -0.59 | 1.76E-09 |
|  |  |  |  | Education | -0.2558 | -0.35 | -0.16 | 7.85E-08 |
|  |  |  |  | SNPxEducation | 0.0029 | -0.08 | 0.08 | 0.94 |
| 2 | 632348 | rs13021737 | A | SNP | -1.4520 | -2.92 | 0.02 | 0.05 |
|  |  |  |  | Age | 0.0525 | 0.03 | 0.07 | 5.51E-09 |
|  |  |  |  | Sex | -0.8799 | -1.17 | -0.59 | 1.70E-09 |
|  |  |  |  | Education | -0.2788 | -0.35 | -0.21 | 1.30E-14 |
|  |  |  |  | SNPxEducation | 0.0736 | -0.03 | 0.18 | 0.16 |
| 2 | 25150296 | rs10182181 | A | SNP | -0.7836 | -1.89 | 0.32 | 0.17 |
|  |  |  |  | Age | 0.0529 | 0.04 | 0.07 | 4.17E-09 |
|  |  |  |  | Sex | -0.8805 | -1.17 | -0.59 | 1.71E-09 |
|  |  |  |  | Education | -0.2949 | -0.40 | -0.19 | 4.25E-08 |
|  |  |  |  | SNPxEducation | 0.0389 | -0.04 | 0.12 | 0.33 |
| 2 | 26928811 | rs11126666 | G | SNP | 0.3292 | -0.99 | 1.65 | 0.62 |
|  |  |  |  | Age | 0.0521 | 0.03 | 0.07 | 7.58E-09 |
|  |  |  |  | Sex | -0.8779 | -1.16 | -0.59 | 1.92E-09 |
|  |  |  |  | Education | -0.1970 | -0.35 | -0.05 | 0.01 |
|  |  |  |  | SNPxEducation | -0.0378 | -0.13 | 0.06 | 0.43 |
| 2 | 59305625 | rs1016287 | C | SNP | -0.5340 | -1.82 | 0.75 | 0.41 |
|  |  |  |  | Age | 0.0528 | 0.04 | 0.07 | 4.49E-09 |
|  |  |  |  | Sex | -0.8857 | -1.17 | -0.60 | 1.41E-09 |
|  |  |  |  | Education | -0.2840 | -0.43 | -0.14 | 1.33E-04 |
|  |  |  |  | SNPxEducation | 0.0211 | -0.07 | 0.11 | 0.65 |
| 2 | 63053048 | rs11688816 | A | SNP | -0.7772 | -1.92 | 0.37 | 0.18 |
|  |  |  |  | Age | 0.0524 | 0.03 | 0.07 | 5.94E-09 |
|  |  |  |  | Sex | -0.8888 | -1.18 | -0.60 | 1.23E-09 |
|  |  |  |  | Education | -0.2957 | -0.39 | -0.20 | 4.10E-09 |
|  |  |  |  | SNPxEducation | 0.0438 | -0.04 | 0.12 | 0.29 |
| 2 | 143043285 | rs2121279 | C | SNP | -0.6192 | -2.23 | 0.99 | 0.45 |
|  |  |  |  | Age | 0.0526 | 0.04 | 0.07 | 5.24E-09 |
|  |  |  |  | Sex | -0.8820 | -1.17 | -0.60 | 1.64E-09 |
|  |  |  |  | Education | -0.3090 | -0.51 | -0.11 | 2.79E-03 |
|  |  |  |  | SNPxEducation | 0.0323 | -0.08 | 0.14 | 0.57 |
| 2 | 164567689 | rs1460676 | T | SNP | -0.4132 | -1.89 | 1.07 | 0.58 |
|  |  |  |  | Age | 0.0525 | 0.03 | 0.07 | 5.77E-09 |
|  |  |  |  | Sex | -0.8826 | -1.17 | -0.60 | 1.61E-09 |
|  |  |  |  | Education | -0.2970 | -0.48 | -0.11 | 0.002 |
|  |  |  |  | SNPxEducation | 0.0265 | -0.08 | 0.13 | 0.62 |
| 2 | 181550962 | rs1528435 | C | SNP | -0.8089 | -1.99 | 0.37 | 0.18 |
|  |  |  |  | Age | 0.0522 | 0.03 | 0.07 | 6.74E-09 |
|  |  |  |  | Sex | -0.8708 | -1.16 | -0.58 | 2.65E-09 |
|  |  |  |  | Education | -0.2855 | -0.37 | -0.20 | 8.62E-11 |
|  |  |  |  | SNPxEducation | 0.0450 | -0.04 | 0.13 | 0.29 |
| 2 | 208255518 | rs17203016 | A | SNP | 0.4517 | -0.99 | 1.89 | 0.54 |
|  |  |  |  | Age | 0.0521 | 0.03 | 0.07 | 7.24E-09 |
|  |  |  |  | Sex | -0.8844 | -1.17 | -0.60 | 1.52E-09 |
|  |  |  |  | Education | -0.1992 | -0.37 | -0.02 | 0.03 |
|  |  |  |  | SNPxEducation | -0.0331 | -0.14 | 0.07 | 0.53 |
| 2 | 213413231 | rs7599312 | A | SNP | -0.9504 | -2.22 | 0.31 | 0.14 |
|  |  |  |  | Age | 0.0522 | 0.03 | 0.07 | 6.97E-09 |
|  |  |  |  | Sex | -0.8866 | -1.17 | -0.60 | 1.36E-09 |
|  |  |  |  | Education | -0.2942 | -0.37 | -0.22 | 2.36E-13 |
|  |  |  |  | SNPxEducation | 0.0748 | -0.01 | 0.16 | 0.10 |
| 2 | 219349752 | rs492400 | T | SNP | -0.2826 | -1.45 | 0.88 | 0.63 |
|  |  |  |  | Age | 0.0524 | 0.03 | 0.07 | 6.15E-09 |
|  |  |  |  | Sex | -0.8818 | -1.17 | -0.60 | 1.71E-09 |
|  |  |  |  | Education | -0.2780 | -0.39 | -0.16 | 1.95E-06 |
|  |  |  |  | SNPxEducation | 0.0203 | -0.06 | 0.10 | 0.63 |
| 2 | 227092802 | rs2176040 | G | SNP | -1.1400 | -2.32 | 0.04 | 0.06 |
|  |  |  |  | Age | 0.0527 | 0.04 | 0.07 | 4.98E-09 |
|  |  |  |  | Sex | -0.8795 | -1.17 | -0.59 | 1.80E-09 |
|  |  |  |  | Education | -0.3538 | -0.48 | -0.23 | 1.37E-08 |
|  |  |  |  | SNPxEducation | 0.0785 | 0.00 | 0.16 | 0.06 |
| 3 | 25106437 | rs6804842 | A | SNP | 0.7464 | -0.39 | 1.88 | 0.20 |
|  |  |  |  | Age | 0.0525 | 0.03 | 0.07 | 5.77E-09 |
|  |  |  |  | Sex | -0.8804 | -1.17 | -0.59 | 1.74E-09 |
|  |  |  |  | Education | -0.2028 | -0.29 | -0.11 | 1.13E-05 |
|  |  |  |  | SNPxEducation | -0.0613 | -0.14 | 0.02 | 0.13 |
| 3 | 61236462 | rs2365389 | T | SNP | 0.2869 | -0.85 | 1.43 | 0.62 |
|  |  |  |  | Age | 0.0524 | 0.03 | 0.07 | 6.01E-09 |
|  |  |  |  | Sex | -0.8791 | -1.17 | -0.59 | 1.86E-09 |
|  |  |  |  | Education | -0.2412 | -0.33 | -0.15 | 2.42E-07 |
|  |  |  |  | SNPxEducation | -0.0142 | -0.09 | 0.07 | 0.73 |
| 3 | 81792112 | rs3849570 | C | SNP | -1.6670 | -2.86 | -0.48 | 0.01 |
|  |  |  |  | Age | 0.0522 | 0.03 | 0.07 | 6.96E-09 |
|  |  |  |  | Sex | -0.8826 | -1.17 | -0.60 | 1.55E-09 |
|  |  |  |  | Education | -0.4004 | -0.53 | -0.27 | 5.60E-10 |
|  |  |  |  | SNPxEducation | 0.1122 | 0.03 | 0.20 | 0.01 |
| 3 | 85807590 | rs13078960 | T | SNP | 0.0469 | -1.39 | 1.49 | 0.95 |
|  |  |  |  | Age | 0.0525 | 0.03 | 0.07 | 5.71E-09 |
|  |  |  |  | Sex | -0.8810 | -1.17 | -0.59 | 1.72E-09 |
|  |  |  |  | Education | -0.2493 | -0.42 | -0.07 | 0.01 |
|  |  |  |  | SNPxEducation | -0.0024 | -0.10 | 0.10 | 0.96 |
| 3 | 141306013 | rs2035935 **†** | G | SNP | 1.0320 | -1.20 | 3.26 | 0.36 |
|  |  |  |  | Age | 0.0526 | 0.03 | 0.07 | 5.43E-09 |
|  |  |  |  | Sex | -0.8783 | -1.16 | -0.59 | 1.91E-09 |
|  |  |  |  | Education | -0.2441 | -0.31 | -0.18 | 1.03E-13 |
|  |  |  |  | SNPxEducation | -0.0631 | -0.22 | 0.10 | 0.44 |
| 3 | 185824004 | rs1516725 | T | SNP | 0.2571 | -1.41 | 1.93 | 0.76 |
|  |  |  |  | Age | 0.0526 | 0.03 | 0.07 | 5.33E-09 |
|  |  |  |  | Sex | -0.8785 | -1.17 | -0.59 | 1.92E-09 |
|  |  |  |  | Education | -0.2504 | -0.32 | -0.18 | 5.62E-13 |
|  |  |  |  | SNPxEducation | -0.0087 | -0.13 | 0.11 | 0.89 |
| 4 | 45182527 | rs10938397 | G | SNP | 0.7462 | -0.40 | 1.90 | 0.20 |
|  |  |  |  | Age | 0.0523 | 0.03 | 0.07 | 6.35E-09 |
|  |  |  |  | Sex | -0.8802 | -1.17 | -0.59 | 1.80E-09 |
|  |  |  |  | Education | -0.2213 | -0.31 | -0.13 | 2.27E-06 |
|  |  |  |  | SNPxEducation | -0.0360 | -0.12 | 0.05 | 0.38 |
| 4 | 77096118 | rs17001561 **†** | A | SNP | -0.0988 | -1.69 | 1.49 | 0.90 |
|  |  |  |  | Age | 0.0526 | 0.03 | 0.07 | 5.44E-09 |
|  |  |  |  | Sex | -0.8798 | -1.17 | -0.59 | 1.81E-09 |
|  |  |  |  | Education | -0.2532 | -0.32 | -0.18 | 1.03E-12 |
|  |  |  |  | SNPxEducation | 0.0013 | -0.11 | 0.11 | 0.98 |
| 4 | 103188709 | rs13107325 | T | SNP | 1.0650 | -1.36 | 3.49 | 0.39 |
|  |  |  |  | Age | 0.0528 | 0.04 | 0.07 | 4.81E-09 |
|  |  |  |  | Sex | -0.8801 | -1.17 | -0.59 | 1.77E-09 |
|  |  |  |  | Education | -0.2462 | -0.31 | -0.18 | 5.68E-14 |
|  |  |  |  | SNPxEducation | -0.0588 | -0.23 | 0.11 | 0.50 |
| 4 | 145659064 | rs11727676 | T | SNP | 0.2673 | -1.69 | 2.22 | 0.79 |
|  |  |  |  | Age | 0.0524 | 0.03 | 0.07 | 6.19E-09 |
|  |  |  |  | Sex | -0.8822 | -1.17 | -0.60 | 1.65E-09 |
|  |  |  |  | Education | -0.2261 | -0.49 | 0.03 | 0.09 |
|  |  |  |  | SNPxEducation | -0.0151 | -0.15 | 0.12 | 0.83 |
| 5 | 75015242 | rs2112347 | G | SNP | -0.2906 | -1.48 | 0.90 | 0.63 |
|  |  |  |  | Age | 0.0526 | 0.03 | 0.07 | 5.39E-09 |
|  |  |  |  | Sex | -0.8803 | -1.17 | -0.59 | 1.77E-09 |
|  |  |  |  | Education | -0.2665 | -0.35 | -0.18 | 1.63E-09 |
|  |  |  |  | SNPxEducation | 0.0181 | -0.07 | 0.10 | 0.67 |
| 5 | 153537893 | rs7715256 | G | SNP | 1.1660 | 0.03 | 2.30 | 0.04 |
|  |  |  |  | Age | 0.0528 | 0.04 | 0.07 | 4.72E-09 |
|  |  |  |  | Sex | -0.8791 | -1.17 | -0.59 | 1.86E-09 |
|  |  |  |  | Education | -0.1864 | -0.28 | -0.10 | 5.43E-05 |
|  |  |  |  | SNPxEducation | -0.0793 | -0.16 | 0.00 | 0.05 |
| 6 | 34563164 | rs205262 | A | SNP | -0.0929 | -1.34 | 1.16 | 0.88 |
|  |  |  |  | Age | 0.0523 | 0.03 | 0.07 | 6.37E-09 |
|  |  |  |  | Sex | -0.8916 | -1.18 | -0.61 | 1.08E-09 |
|  |  |  |  | Education | -0.2373 | -0.38 | -0.10 | 0.001 |
|  |  |  |  | SNPxEducation | -0.0126 | -0.10 | 0.08 | 0.78 |
| 6 | 40348653 | rs2033529 | G | SNP | 0.3228 | -0.94 | 1.58 | 0.62 |
|  |  |  |  | Age | 0.0524 | 0.03 | 0.07 | 6.02E-09 |
|  |  |  |  | Sex | -0.8796 | -1.17 | -0.59 | 1.82E-09 |
|  |  |  |  | Education | -0.2411 | -0.32 | -0.16 | 4.69E-09 |
|  |  |  |  | SNPxEducation | -0.0203 | -0.11 | 0.07 | 0.65 |
| 6 | 50845490 | rs2207139 | G | SNP | 0.8447 | -0.62 | 2.31 | 0.26 |
|  |  |  |  | Age | 0.0540 | 0.04 | 0.07 | 1.98E-09 |
|  |  |  |  | Sex | -0.8680 | -1.15 | -0.58 | 2.80E-09 |
|  |  |  |  | Education | -0.2414 | -0.31 | -0.17 | 3.63E-11 |
|  |  |  |  | SNPxEducation | -0.0252 | -0.13 | 0.08 | 0.63 |
| 6 | 108977663 | rs9400239 | T | SNP | -1.2770 | -2.53 | -0.03 | 0.05 |
|  |  |  |  | Age | 0.0524 | 0.03 | 0.07 | 6.01E-09 |
|  |  |  |  | Sex | -0.8916 | -1.18 | -0.61 | 1.08E-09 |
|  |  |  |  | Education | -0.2970 | -0.38 | -0.22 | 1.91E-13 |
|  |  |  |  | SNPxEducation | 0.0738 | -0.01 | 0.16 | 0.10 |
| 6 | 120185665 | rs9374842 | C | SNP | 0.0678 | -1.27 | 1.40 | 0.92 |
|  |  |  |  | Age | 0.0525 | 0.03 | 0.07 | 5.52E-09 |
|  |  |  |  | Sex | -0.8838 | -1.17 | -0.60 | 1.51E-09 |
|  |  |  |  | Education | -0.2488 | -0.33 | -0.17 | 2.88E-10 |
|  |  |  |  | SNPxEducation | -0.0087 | -0.10 | 0.09 | 0.86 |
| 6 | 137675541 | rs13201877 | A | SNP | 0.4363 | -1.19 | 2.06 | 0.60 |
|  |  |  |  | Age | 0.0527 | 0.04 | 0.07 | 5.02E-09 |
|  |  |  |  | Sex | -0.8793 | -1.17 | -0.59 | 1.85E-09 |
|  |  |  |  | Education | -0.1956 | -0.40 | 0.01 | 0.06 |
|  |  |  |  | SNPxEducation | -0.0337 | -0.15 | 0.08 | 0.57 |
| 6 | 163033350 | rs13191362 | G | SNP | -0.8305 | -2.71 | 1.05 | 0.39 |
|  |  |  |  | Age | 0.0524 | 0.03 | 0.07 | 6.34E-09 |
|  |  |  |  | Sex | -0.8766 | -1.16 | -0.59 | 2.06E-09 |
|  |  |  |  | Education | -0.2680 | -0.33 | -0.20 | 4.75E-15 |
|  |  |  |  | SNPxEducation | 0.0691 | -0.06 | 0.20 | 0.31 |
| 7 | 75163169 | rs1167827 | G | SNP | 0.9739 | -0.17 | 2.12 | 0.10 |
|  |  |  |  | Age | 0.0526 | 0.03 | 0.07 | 5.32E-09 |
|  |  |  |  | Sex | -0.8889 | -1.18 | -0.60 | 1.23E-09 |
|  |  |  |  | Education | -0.1812 | -0.29 | -0.07 | 0.001 |
|  |  |  |  | SNPxEducation | -0.0625 | -0.14 | 0.02 | 0.13 |
| 7 | 76608143 | rs2245368 | C | SNP | 0.4518 | -1.09 | 1.99 | 0.57 |
|  |  |  |  | Age | 0.0519 | 0.03 | 0.07 | 8.68E-09 |
|  |  |  |  | Sex | -0.8814 | -1.17 | -0.60 | 1.67E-09 |
|  |  |  |  | Education | -0.2478 | -0.32 | -0.18 | 6.29E-12 |
|  |  |  |  | SNPxEducation | -0.0212 | -0.13 | 0.09 | 0.70 |
| 7 | 93568420 | rs9641123 | C | SNP | -0.3987 | -1.63 | 0.84 | 0.52 |
|  |  |  |  | Age | 0.0548 | 0.04 | 0.74 | 1.20E-08 |
|  |  |  |  | Sex | -1.0337 | -1.34 | -0.73 | 3.96E-11 |
|  |  |  |  | Education | -0.2830 | -0.38 | -0.19 | 1.14E-08 |
|  |  |  |  | SNPxEducation | 0.0333 | -0.05 | 0.12 | 0.45 |
| 7 | 95169514 | rs6465468 | T | SNP | 0.2028 | -1.02 | 1.43 | 0.75 |
|  |  |  |  | Age | 0.0523 | 0.03 | 0.07 | 6.50E-09 |
|  |  |  |  | Sex | -0.8829 | -1.17 | -0.60 | 1.58E-09 |
|  |  |  |  | Education | -0.2394 | -0.32 | -0.16 | 5.98E-09 |
|  |  |  |  | SNPxEducation | -0.0223 | -0.11 | 0.06 | 0.61 |
| 8 | 76806584 | rs17405819 | T | SNP | 0.4138 | -0.84 | 1.67 | 0.52 |
|  |  |  |  | Age | 0.0525 | 0.03 | 0.07 | 5.69E-09 |
|  |  |  |  | Sex | -0.8849 | -1.17 | -0.60 | 1.45E-09 |
|  |  |  |  | Education | -0.2278 | -0.37 | -0.09 | 0.001 |
|  |  |  |  | SNPxEducation | -0.0182 | -0.11 | 0.07 | 0.69 |
| 8 | 81375457 | rs16907751 | C | SNP | -0.0687 | -1.87 | 1.73 | 0.94 |
|  |  |  |  | Age | 0.0525 | 0.03 | 0.07 | 5.69E-09 |
|  |  |  |  | Sex | -0.8808 | -1.17 | -0.59 | 1.76E-09 |
|  |  |  |  | Education | -0.2666 | -0.50 | -0.04 | 0.02 |
|  |  |  |  | SNPxEducation | 0.0076 | -0.12 | 0.13 | 0.91 |
| 8 | 85079709 | rs2033732 | T | SNP | 0.5783 | -0.71 | 1.87 | 0.38 |
|  |  |  |  | Age | 0.0525 | 0.03 | 0.07 | 5.78E-09 |
|  |  |  |  | Sex | -0.8787 | -1.17 | -0.59 | 1.89E-09 |
|  |  |  |  | Education | -0.2354 | -0.31 | -0.16 | 1.42E-09 |
|  |  |  |  | SNPxEducation | -0.0341 | -0.13 | 0.06 | 0.46 |
| 9 | 15634326 | rs4740619 | T | SNP | 0.3127 | -0.83 | 1.46 | 0.59 |
|  |  |  |  | Age | 0.0524 | 0.03 | 0.07 | 6.19E-09 |
|  |  |  |  | Sex | -0.8823 | -1.17 | -0.60 | 1.63E-09 |
|  |  |  |  | Education | -0.2288 | -0.34 | -0.12 | 4.15E-05 |
|  |  |  |  | SNPxEducation | -0.0217 | -0.10 | 0.06 | 0.60 |
| 9 | 28414339 | rs10968576 | G | SNP | 0.8892 | -0.34 | 2.12 | 0.16 |
|  |  |  |  | Age | 0.0529 | 0.04 | 0.07 | 4.40E-09 |
|  |  |  |  | Sex | -0.8765 | -1.16 | -0.59 | 2.07E-09 |
|  |  |  |  | Education | -0.2163 | -0.30 | -0.13 | 2.28E-07 |
|  |  |  |  | SNPxEducation | -0.0569 | -0.14 | 0.03 | 0.20 |
| 9 | 111932342 | rs6477694 | C | SNP | -0.3344 | -1.53 | 0.86 | 0.58 |
|  |  |  |  | Age | 0.0525 | 0.03 | 0.07 | 5.72E-09 |
|  |  |  |  | Sex | -0.8812 | -1.17 | -0.60 | 1.69E-09 |
|  |  |  |  | Education | -0.2706 | -0.36 | -0.18 | 1.06E-09 |
|  |  |  |  | SNPxEducation | 0.0235 | -0.06 | 0.11 | 0.58 |
| 9 | 120378483 | rs1928295 | C | SNP | -1.4850 | -2.60 | -0.37 | 0.01 |
|  |  |  |  | Age | 0.0525 | 0.03 | 0.07 | 5.72E-09 |
|  |  |  |  | Sex | -0.8776 | -1.16 | -0.59 | 1.91E-09 |
|  |  |  |  | Education | -0.3449 | -0.44 | -0.25 | 1.69E-12 |
|  |  |  |  | SNPxEducation | 0.0973 | 0.02 | 0.18 | 0.02 |
| 9 | 129460914 | rs10733682 | A | SNP | -0.9055 | -2.05 | 0.24 | 0.12 |
|  |  |  |  | Age | 0.0524 | 0.03 | 0.07 | 6.15E-09 |
|  |  |  |  | Sex | -0.8885 | -1.18 | -0.60 | 1.26E-09 |
|  |  |  |  | Education | -0.3067 | -0.41 | -0.21 | 1.87E-09 |
|  |  |  |  | SNPxEducation | 0.0538 | -0.03 | 0.13 | 0.19 |
| 10 | 87410904 | rs7899106 | G | SNP | -1.8630 | -4.56 | 0.83 | 0.18 |
|  |  |  |  | Age | 0.0525 | 0.03 | 0.07 | 5.75E-09 |
|  |  |  |  | Sex | -0.8799 | -1.17 | -0.59 | 1.77E-09 |
|  |  |  |  | Education | -0.2660 | -0.33 | -0.20 | 2.12E-16 |
|  |  |  |  | SNPxEducation | 0.1492 | -0.04 | 0.34 | 0.13 |
| 10 | 102395440 | rs17094222 | T | SNP | 0.4122 | -1.04 | 1.86 | 0.58 |
|  |  |  |  | Age | 0.0527 | 0.04 | 0.07 | 5.15E-09 |
|  |  |  |  | Sex | -0.8786 | -1.17 | -0.59 | 1.92E-09 |
|  |  |  |  | Education | -0.1929 | -0.37 | -0.02 | 0.03 |
|  |  |  |  | SNPxEducation | -0.0370 | -0.14 | 0.07 | 0.48 |
| 10 | 104869038 | rs11191560 | C | SNP | 0.4089 | -1.43 | 2.24 | 0.66 |
|  |  |  |  | Age | 0.0525 | 0.03 | 0.07 | 5.73E-09 |
|  |  |  |  | Sex | -0.8806 | -1.17 | -0.59 | 1.74E-09 |
|  |  |  |  | Education | -0.2487 | -0.32 | -0.18 | 2.60E-13 |
|  |  |  |  | SNPxEducation | -0.0220 | -0.15 | 0.11 | 0.74 |
| 10 | 114758349 | rs7903146 | T | SNP | -0.9586 | -2.22 | 0.30 | 0.14 |
|  |  |  |  | Age | 0.0524 | 0.03 | 0.07 | 6.06E-09 |
|  |  |  |  | Sex | -0.8824 | -1.17 | -0.60 | 1.61E-09 |
|  |  |  |  | Education | -0.2851 | -0.36 | -0.21 | 8.38E-13 |
|  |  |  |  | SNPxEducation | 0.0587 | -0.03 | 0.15 | 0.19 |
| 11 | 8673939 | rs4256980 | C | SNP | 0.7779 | -0.38 | 1.94 | 0.19 |
|  |  |  |  | Age | 0.0528 | 0.04 | 0.07 | 4.55E-09 |
|  |  |  |  | Sex | -0.8774 | -1.16 | -0.59 | 1.98E-09 |
|  |  |  |  | Education | -0.2073 | -0.29 | -0.12 | 2.50E-06 |
|  |  |  |  | SNPxEducation | -0.0612 | -0.14 | 0.02 | 0.14 |
| 11 | 27684517 | rs11030104 | G | SNP | -1.8070 | -3.23 | -0.39 | 0.01 |
|  |  |  |  | Age | 0.0524 | 0.03 | 0.07 | 5.75E-09 |
|  |  |  |  | Sex | -0.8851 | -1.17 | -0.60 | 1.37E-09 |
|  |  |  |  | Education | -0.2962 | -0.37 | -0.22 | 7.80E-15 |
|  |  |  |  | SNPxEducation | 0.1016 | 0.00 | 0.20 | 0.05 |
| 11 | 43864278 | rs2176598 | T | SNP | 0.5225 | -0.77 | 1.82 | 0.43 |
|  |  |  |  | Age | 0.0524 | 0.03 | 0.07 | 6.03E-09 |
|  |  |  |  | Sex | -0.8832 | -1.17 | -0.60 | 1.57E-09 |
|  |  |  |  | Education | -0.2376 | -0.31 | -0.16 | 6.91E-10 |
|  |  |  |  | SNPxEducation | -0.0312 | -0.12 | 0.06 | 0.50 |
| 11 | 47650993 | rs3817334 | T | SNP | -0.4472 | -1.60 | 0.70 | 0.45 |
|  |  |  |  | Age | 0.0527 | 0.04 | 0.07 | 5.09E-09 |
|  |  |  |  | Sex | -0.8757 | -1.16 | -0.59 | 2.20E-09 |
|  |  |  |  | Education | -0.2801 | -0.37 | -0.19 | 9.86E-10 |
|  |  |  |  | SNPxEducation | 0.0331 | -0.05 | 0.11 | 0.42 |
| 11 | 115022404 | rs12286929 | A | SNP | 0.2642 | -0.90 | 1.43 | 0.66 |
|  |  |  |  | Age | 0.0525 | 0.03 | 0.07 | 5.61E-09 |
|  |  |  |  | Sex | -0.8844 | -1.17 | -0.60 | 1.47E-09 |
|  |  |  |  | Education | -0.2233 | -0.32 | -0.13 | 5.39E-06 |
|  |  |  |  | SNPxEducation | -0.0321 | -0.11 | 0.05 | 0.44 |
| 12 | 50247468 | rs7138803 | G | SNP | -0.3150 | -1.47 | 0.84 | 0.59 |
|  |  |  |  | Age | 0.0526 | 0.03 | 0.07 | 5.34E-09 |
|  |  |  |  | Sex | -0.8763 | -1.16 | -0.59 | 2.07E-09 |
|  |  |  |  | Education | -0.2638 | -0.38 | -0.15 | 7.05E-06 |
|  |  |  |  | SNPxEducation | 0.0087 | -0.07 | 0.09 | 0.83 |
| 12 | 122781897 | rs11057405 | G | SNP | 2.6240 | 0.79 | 4.46 | 0.01 |
|  |  |  |  | Age | 0.0518 | 0.03 | 0.07 | 8.67E-09 |
|  |  |  |  | Sex | -0.8855 | -1.17 | -0.60 | 1.38E-09 |
|  |  |  |  | Education | 0.0427 | -0.19 | 0.28 | 0.72 |
|  |  |  |  | SNPxEducation | -0.1650 | -0.29 | -0.04 | 0.01 |
| 13 | 26915782 | rs9581855 **†** | A | SNP | 0.2646 | -1.23 | 1.76 | 0.72 |
|  |  |  |  | Age | 0.0547 | 0.04 | 0.07 | 1.24E-08 |
|  |  |  |  | Sex | -1.0314 | -1.34 | -0.73 | 4.22E-11 |
|  |  |  |  | Education | -0.2551 | -0.33 | -0.18 | 1.57E-10 |
|  |  |  |  | SNPxEducation | 0.0032 | -0.10 | 0.11 | 0.95 |
| 13 | 54102206 | rs12429545 | G | SNP | -0.9046 | -2.63 | 0.82 | 0.31 |
|  |  |  |  | Age | 0.0527 | 0.04 | 0.07 | 4.97E-09 |
|  |  |  |  | Sex | -0.8866 | -1.17 | -0.60 | 1.37E-09 |
|  |  |  |  | Education | -0.3357 | -0.55 | -0.12 | 0.003 |
|  |  |  |  | SNPxEducation | 0.0467 | -0.07 | 0.17 | 0.45 |
| 13 | 66205704 | rs9540493 | A | SNP | 1.4540 | 0.33 | 2.58 | 0.01 |
|  |  |  |  | Age | 0.0528 | 0.04 | 0.07 | 4.46E-09 |
|  |  |  |  | Sex | -0.8783 | -1.16 | -0.59 | 1.83E-09 |
|  |  |  |  | Education | -0.1800 | -0.27 | -0.09 | 1.16E-04 |
|  |  |  |  | SNPxEducation | -0.0861 | -0.17 | -0.01 | 0.03 |
| 13 | 79580919 | rs1441264 | A | SNP | 0.2903 | -0.89 | 1.47 | 0.63 |
|  |  |  |  | Age | 0.0525 | 0.03 | 0.07 | 5.71E-09 |
|  |  |  |  | Sex | -0.8788 | -1.17 | -0.59 | 1.89E-09 |
|  |  |  |  | Education | -0.2308 | -0.35 | -0.11 | 1.21E-04 |
|  |  |  |  | SNPxEducation | -0.0182 | -0.10 | 0.06 | 0.67 |
| 14 | 25928179 | rs10132280 | A | SNP | 0.9080 | -0.35 | 2.17 | 0.16 |
|  |  |  |  | Age | 0.0526 | 0.03 | 0.07 | 5.28E-09 |
|  |  |  |  | Sex | -0.8783 | -1.16 | -0.59 | 1.93E-09 |
|  |  |  |  | Education | -0.2169 | -0.30 | -0.14 | 1.20E-07 |
|  |  |  |  | SNPxEducation | -0.0617 | -0.15 | 0.03 | 0.17 |
| 14 | 29736838 | rs12885454 | C | SNP | 1.1600 | -0.03 | 2.35 | 0.06 |
|  |  |  |  | Age | 0.0521 | 0.03 | 0.07 | 7.20E-09 |
|  |  |  |  | Sex | -0.8826 | -1.17 | -0.60 | 1.58E-09 |
|  |  |  |  | Education | -0.1539 | -0.28 | -0.03 | 0.02 |
|  |  |  |  | SNPxEducation | -0.0747 | -0.16 | 0.01 | 0.08 |
| 14 | 30515112 | rs11847697 | T | SNP | 0.2920 | -2.60 | 3.18 | 0.84 |
|  |  |  |  | Age | 0.0526 | 0.04 | 0.07 | 5.21E-09 |
|  |  |  |  | Sex | -0.8796 | -1.17 | -0.59 | 1.83E-09 |
|  |  |  |  | Education | -0.2530 | -0.32 | -0.19 | 5.26E-15 |
|  |  |  |  | SNPxEducation | 0.0014 | -0.20 | 0.21 | 0.99 |
| 14 | 79899454 | rs7141420 | C | SNP | -0.6532 | -1.80 | 0.49 | 0.26 |
|  |  |  |  | Age | 0.0523 | 0.03 | 0.07 | 6.58E-09 |
|  |  |  |  | Sex | -0.8830 | -1.17 | -0.60 | 1.57E-09 |
|  |  |  |  | Education | -0.3016 | -0.40 | -0.20 | 1.67E-09 |
|  |  |  |  | SNPxEducation | 0.0509 | -0.03 | 0.13 | 0.22 |
| 15 | 51748610 | rs3736485 | A | SNP | 0.4330 | -0.69 | 1.56 | 0.45 |
|  |  |  |  | Age | 0.0525 | 0.03 | 0.07 | 5.52E-09 |
|  |  |  |  | Sex | -0.8804 | -1.17 | -0.59 | 1.75E-09 |
|  |  |  |  | Education | -0.2269 | -0.32 | -0.13 | 1.59E-06 |
|  |  |  |  | SNPxEducation | -0.0299 | -0.11 | 0.05 | 0.46 |
| 15 | 68077168 | rs16951275 | C | SNP | -0.1454 | -1.49 | 1.20 | 0.83 |
|  |  |  |  | Age | 0.0524 | 0.03 | 0.07 | 5.99E-09 |
|  |  |  |  | Sex | -0.8818 | -1.17 | -0.60 | 1.66E-09 |
|  |  |  |  | Education | -0.2550 | -0.33 | -0.18 | 3.71E-11 |
|  |  |  |  | SNPxEducation | 0.0045 | -0.09 | 0.10 | 0.93 |
| 15 | 73093991 | rs7164727 | C | SNP | -0.7829 | -2.00 | 0.44 | 0.21 |
|  |  |  |  | Age | 0.0526 | 0.03 | 0.07 | 5.33E-09 |
|  |  |  |  | Sex | -0.8833 | -1.17 | -0.60 | 1.54E-09 |
|  |  |  |  | Education | -0.2833 | -0.36 | -0.20 | 7.54E-12 |
|  |  |  |  | SNPxEducation | 0.0478 | -0.04 | 0.13 | 0.28 |
| 16 | 3627358 | rs758747 | T | SNP | 1.3550 | 0.06 | 2.65 | 0.04 |
|  |  |  |  | Age | 0.0523 | 0.03 | 0.07 | 6.30E-09 |
|  |  |  |  | Sex | -0.8787 | -1.17 | -0.59 | 1.85E-09 |
|  |  |  |  | Education | -0.2048 | -0.28 | -0.13 | 2.87E-07 |
|  |  |  |  | SNPxEducation | -0.0898 | -0.18 | 0.00 | 0.05 |
| 16 | 19935389 | rs12446632 | A | SNP | -0.0762 | -1.72 | 1.57 | 0.93 |
|  |  |  |  | Age | 0.0527 | 0.04 | 0.07 | 5.23E-09 |
|  |  |  |  | Sex | -0.8904 | -1.18 | -0.60 | 1.19E-09 |
|  |  |  |  | Education | -0.2534 | -0.32 | -0.18 | 6.11E-13 |
|  |  |  |  | SNPxEducation | -0.0041 | -0.12 | 0.11 | 0.95 |
| 16 | 28333411 | rs2650492 | A | SNP | 0.1317 | -1.09 | 1.35 | 0.83 |
|  |  |  |  | Age | 0.0525 | 0.03 | 0.07 | 5.88E-09 |
|  |  |  |  | Sex | -0.8801 | -1.17 | -0.59 | 1.78E-09 |
|  |  |  |  | Education | -0.2449 | -0.33 | -0.16 | 3.78E-09 |
|  |  |  |  | SNPxEducation | -0.0131 | -0.10 | 0.07 | 0.77 |
| 16 | 28889486 | rs3888190 | A | SNP | 0.4887 | -0.69 | 1.67 | 0.42 |
|  |  |  |  | Age | 0.0524 | 0.03 | 0.07 | 6.13E-09 |
|  |  |  |  | Sex | -0.8821 | -1.17 | -0.60 | 1.66E-09 |
|  |  |  |  | Education | -0.2251 | -0.31 | -0.14 | 7.62E-07 |
|  |  |  |  | SNPxEducation | -0.0359 | -0.12 | 0.05 | 0.40 |
| 16 | 30015337 | rs4787491 | A | SNP | -0.8347 | -1.97 | 0.30 | 0.15 |
|  |  |  |  | Age | 0.0525 | 0.03 | 0.07 | 5.66E-09 |
|  |  |  |  | Sex | -0.8770 | -1.16 | -0.59 | 2.02E-09 |
|  |  |  |  | Education | -0.3100 | -0.41 | -0.21 | 5.58E-10 |
|  |  |  |  | SNPxEducation | 0.0593 | -0.02 | 0.14 | 0.15 |
| 16 | 31129895 | rs9925964 | G | SNP | -0.5425 | -1.72 | 0.63 | 0.37 |
|  |  |  |  | Age | 0.0524 | 0.03 | 0.07 | 6.25E-09 |
|  |  |  |  | Sex | -0.8836 | -1.17 | -0.60 | 1.54E-09 |
|  |  |  |  | Education | -0.2806 | -0.37 | -0.19 | 3.98E-10 |
|  |  |  |  | SNPxEducation | 0.0361 | -0.05 | 0.12 | 0.39 |
| 16 | 49062590 | rs2080454 | C | SNP | -0.5674 | -1.74 | 0.61 | 0.34 |
|  |  |  |  | Age | 0.0526 | 0.03 | 0.07 | 5.33E-09 |
|  |  |  |  | Sex | -0.8780 | -1.16 | -0.59 | 1.93E-09 |
|  |  |  |  | Education | -0.2895 | -0.38 | -0.20 | 9.91E-11 |
|  |  |  |  | SNPxEducation | 0.0484 | -0.03 | 0.13 | 0.25 |
| 16 | 53803574 | rs1558902 | A | SNP | 0.4881 | -0.65 | 1.63 | 0.40 |
|  |  |  |  | Age | 0.0520 | 0.03 | 0.07 | 7.81E-09 |
|  |  |  |  | Sex | -0.8727 | -1.16 | -0.59 | 2.37E-09 |
|  |  |  |  | Education | -0.2448 | -0.34 | -0.15 | 1.90E-07 |
|  |  |  |  | SNPxEducation | -0.0113 | -0.09 | 0.07 | 0.78 |
| 17 | 2005136 | rs9914578 | G | SNP | 0.4714 | -0.91 | 1.85 | 0.50 |
|  |  |  |  | Age | 0.0524 | 0.03 | 0.07 | 6.12E-09 |
|  |  |  |  | Sex | -0.8780 | -1.16 | -0.59 | 1.97E-09 |
|  |  |  |  | Education | -0.2391 | -0.31 | -0.17 | 1.39E-10 |
|  |  |  |  | SNPxEducation | -0.0330 | -0.13 | 0.06 | 0.51 |
| 17 | 5283252 | rs1000940 | G | SNP | -0.1352 | -1.38 | 1.11 | 0.83 |
|  |  |  |  | Age | 0.0529 | 0.04 | 0.07 | 4.42E-09 |
|  |  |  |  | Sex | -0.8778 | -1.16 | -0.59 | 1.94E-09 |
|  |  |  |  | Education | -0.2660 | -0.35 | -0.19 | 9.36E-11 |
|  |  |  |  | SNPxEducation | 0.0236 | -0.06 | 0.11 | 0.60 |
| 17 | 78615571 | rs12940622 | A | SNP | -1.4620 | -2.61 | -0.31 | 0.01 |
|  |  |  |  | Age | 0.0528 | 0.04 | 0.07 | 4.49E-09 |
|  |  |  |  | Sex | -0.8843 | -1.17 | -0.60 | 1.48E-09 |
|  |  |  |  | Education | -0.3382 | -0.43 | -0.24 | 2.33E-12 |
|  |  |  |  | SNPxEducation | 0.0955 | 0.01 | 0.18 | 0.02 |
| 18 | 21104888 | rs1808579 | T | SNP | 0.1731 | -0.95 | 1.30 | 0.76 |
|  |  |  |  | Age | 0.0526 | 0.03 | 0.07 | 5.44E-09 |
|  |  |  |  | Sex | -0.8783 | -1.16 | -0.59 | 1.92E-09 |
|  |  |  |  | Education | -0.2409 | -0.34 | -0.14 | 1.27E-06 |
|  |  |  |  | SNPxEducation | -0.0133 | -0.09 | 0.07 | 0.74 |
| 18 | 40147671 | rs7239883 | G | SNP | -0.3462 | -1.51 | 0.82 | 0.56 |
|  |  |  |  | Age | 0.0527 | 0.04 | 0.07 | 5.11E-09 |
|  |  |  |  | Sex | -0.8834 | -1.17 | -0.60 | 1.56E-09 |
|  |  |  |  | Education | -0.2784 | -0.37 | -0.19 | 8.01E-10 |
|  |  |  |  | SNPxEducation | 0.0329 | -0.05 | 0.11 | 0.43 |
| 18 | 56883319 | rs7243357 | G | SNP | 0.3902 | -1.14 | 1.93 | 0.62 |
|  |  |  |  | Age | 0.0524 | 0.03 | 0.07 | 6.02E-09 |
|  |  |  |  | Sex | -0.8811 | -1.17 | -0.59 | 1.72E-09 |
|  |  |  |  | Education | -0.2455 | -0.32 | -0.17 | 1.14E-11 |
|  |  |  |  | SNPxEducation | -0.0231 | -0.13 | 0.08 | 0.67 |
| 18 | 57829135 | rs6567160 | C | SNP | -0.7841 | -2.09 | 0.53 | 0.24 |
|  |  |  |  | Age | 0.0526 | 0.03 | 0.07 | 5.24E-09 |
|  |  |  |  | Sex | -0.8856 | -1.17 | -0.60 | 1.35E-09 |
|  |  |  |  | Education | -0.2920 | -0.37 | -0.22 | 7.08E-14 |
|  |  |  |  | SNPxEducation | 0.0752 | -0.02 | 0.17 | 0.11 |
| 19 | 18454825 | rs17724992 | G | SNP | -0.1767 | -1.51 | 1.15 | 0.79 |
|  |  |  |  | Age | 0.0525 | 0.03 | 0.07 | 5.81E-09 |
|  |  |  |  | Sex | -0.8825 | -1.17 | -0.60 | 1.62E-09 |
|  |  |  |  | Education | -0.2570 | -0.33 | -0.18 | 7.18E-11 |
|  |  |  |  | SNPxEducation | 0.0074 | -0.09 | 0.10 | 0.88 |
| 19 | 34309532 | rs29941 | A | SNP | 0.6575 | -0.53 | 1.84 | 0.28 |
|  |  |  |  | Age | 0.0525 | 0.03 | 0.07 | 5.48E-09 |
|  |  |  |  | Sex | -0.8827 | -1.17 | -0.60 | 1.60E-09 |
|  |  |  |  | Education | -0.2202 | -0.30 | -0.14 | 1.14E-07 |
|  |  |  |  | SNPxEducation | -0.0510 | -0.13 | 0.03 | 0.23 |
| 19 | 45395619 | rs2075650 | G | SNP | -0.8359 | -2.43 | 0.76 | 0.30 |
|  |  |  |  | Age | 0.0525 | 0.03 | 0.07 | 5.80E-09 |
|  |  |  |  | Sex | -0.8821 | -1.17 | -0.60 | 1.64E-09 |
|  |  |  |  | Education | -0.2725 | -0.34 | -0.20 | 2.13E-14 |
|  |  |  |  | SNPxEducation | 0.0637 | -0.05 | 0.18 | 0.27 |
| 19 | 46202172 | rs2287019 | T | SNP | 0.8671 | -0.55 | 2.29 | 0.23 |
|  |  |  |  | Age | 0.0522 | 0.03 | 0.07 | 6.70E-09 |
|  |  |  |  | Sex | -0.8766 | -1.16 | -0.59 | 1.98E-09 |
|  |  |  |  | Education | -0.2175 | -0.29 | -0.14 | 5.52E-09 |
|  |  |  |  | SNPxEducation | -0.0850 | -0.18 | 0.01 | 0.10 |
| 19 | 47569003 | rs3810291 | G | SNP | -1.1160 | -2.35 | 0.12 | 0.08 |
|  |  |  |  | Age | 0.0521 | 0.03 | 0.07 | 7.15E-09 |
|  |  |  |  | Sex | -0.8768 | -1.16 | -0.59 | 2.02E-09 |
|  |  |  |  | Education | -0.2929 | -0.37 | -0.21 | 7.75E-13 |
|  |  |  |  | SNPxEducation | 0.0663 | -0.02 | 0.15 | 0.14 |
| 20 | 51087862 | rs6091540 | T | SNP | -0.4380 | -1.75 | 0.88 | 0.51 |
|  |  |  |  | Age | 0.0526 | 0.03 | 0.07 | 5.32E-09 |
|  |  |  |  | Sex | -0.8807 | -1.17 | -0.59 | 1.73E-09 |
|  |  |  |  | Education | -0.2641 | -0.34 | -0.19 | 1.19E-11 |
|  |  |  |  | SNPxEducation | 0.0224 | -0.07 | 0.11 | 0.63 |
| 21 | 40291740 | rs2836754 | T | SNP | -0.2263 | -1.40 | 0.95 | 0.71 |
|  |  |  |  | Age | 0.0526 | 0.03 | 0.07 | 5.31E-09 |
|  |  |  |  | Sex | -0.8821 | -1.17 | -0.60 | 1.64E-09 |
|  |  |  |  | Education | -0.2624 | -0.35 | -0.18 | 3.00E-09 |
|  |  |  |  | SNPxEducation | 0.0124 | -0.07 | 0.10 | 0.77 |

*Position of build 36; † proxy SNPs (rs2035935 as a proxy for rs16851483, rs17001561 as a proxy for rs17001654 and rs9581855 as a proxy for rs12016871), LD of proxy SNPs r²>0.9.
