# Supplementary material for: A genetic sum score of risk alleles associated with body mass index interacts with socioeconomic position in the Heinz Nixdorf Recall Study
Source: PLoS One. 2019 Aug 23;14(8):e0221252. doi: 10.1371/journal.pone.0221252 (PMC6707579; doi:10.1371/journal.pone.0221252)
Supplement: S2 Table — (DOCX) [file pone.0221252.s002.docx]

**S2 Table. Sex- and age-adjusted effects and corresponding 95% confidence interval (95% CI) of the interaction of each body mass index (BMI)- associated single-nucleotide polymorphism (SNP) with income (per 1000€) on BMI.**

| **CHR** | **Position*** | **SNP** | **Risk Allele** | **TEST** | **BETA** | **95 % CI** | | **P** |
| --- | --- | --- | --- | --- | --- | --- | --- | --- |
|  |  |  |  |  |  | **Lower** | **Upper** |  |
| 1 | 47684677 | rs977747 | G | SNP | -0.5531 | -1.06 | -0.05 | 0.03 |
|  |  |  |  | Age | 0.0586 | 0.04 | 0.08 | 1.63E-10 |
|  |  |  |  | Sex | -0.5593 | -0.84 | -0.28 | 9.65E-05 |
|  |  |  |  | Income | -0.9036 | -1.32 | -0.49 | 2.43E-05 |
|  |  |  |  | SNPxIncome | 0.2507 | -0.04 | 0.54 | 0.09 |
| 1 | 49589847 | rs657452 | G | SNP | 0.0423 | -0.45 | 0.53 | 0.87 |
|  |  |  |  | Age | 0.0573 | 0.04 | 0.08 | 5.10E-10 |
|  |  |  |  | Sex | -0.5564 | -0.84 | -0.27 | 1.15E-04 |
|  |  |  |  | Income | -0.4804 | -0.89 | -0.07 | 0.02119 |
|  |  |  |  | SNPxIncome | -0.0855 | -0.37 | 0.20 | 0.55 |
| 1 | 50559820 | rs11583200 | T | SNP | -0.0105 | -0.51 | 0.49 | 0.97 |
|  |  |  |  | Age | 0.0586 | 0.04 | 0.08 | 1.67E-10 |
|  |  |  |  | Sex | -0.5582 | -0.84 | -0.28 | 1.01E-04 |
|  |  |  |  | Income | -0.5666 | -0.98 | -0.15 | 0.01 |
|  |  |  |  | SNPxIncome | -0.0112 | -0.30 | 0.28 | 0.94 |
| 1 | 72751185 | rs3101336 | T | SNP | -0.4560 | -0.96 | 0.05 | 0.08 |
|  |  |  |  | Age | 0.0587 | 0.04 | 0.08 | 1.49E-10 |
|  |  |  |  | Sex | -0.5560 | -0.84 | -0.28 | 1.06E-04 |
|  |  |  |  | Income | -0.6872 | -0.98 | -0.39 | 5.89E-06 |
|  |  |  |  | SNPxIncome | 0.1411 | -0.15 | 0.43 | 0.34 |
| 1 | 75002193 | rs12566985 | A | SNP | -0.1203 | -0.61 | 0.37 | 0.63 |
|  |  |  |  | Age | 0.0588 | 0.04 | 0.08 | 1.50E-10 |
|  |  |  |  | Sex | -0.5541 | -0.84 | -0.27 | 1.15E-04 |
|  |  |  |  | Income | -0.6172 | -1.00 | -0.23 | 0.002 |
|  |  |  |  | SNPxIncome | 0.0178 | -0.27 | 0.31 | 0.90 |
| 1 | 78446761 | rs12401738 | G | SNP | -0.1042 | -0.61 | 0.4 | 0.69 |
|  |  |  |  | Age | 0.0587 | 0.04 | 0.08 | 1.53E-10 |
|  |  |  |  | Sex | -0.5586 | -0.84 | -0.28 | 9.91E-05 |
|  |  |  |  | Income | -0.5460 | -0.98 | -0.11 | 0.01336 |
|  |  |  |  | SNPxIncome | -0.0266 | -0.32 | 0.27 | 0.86 |
| 1 | 96924097 | rs11165643 | C | SNP | -0.3287 | -0.82 | 0.16 | 0.19 |
|  |  |  |  | Age | 0.0590 | 0.04 | 0.08 | 1.28E-10 |
|  |  |  |  | Sex | -0.5583 | -0.84 | -0.28 | 1.00E-04 |
|  |  |  |  | Income | -0.6559 | -0.96 | -0.35 | 3.16E-05 |
|  |  |  |  | SNPxIncome | 0.0934 | -0.19 | 0.38 | 0.52 |
| 1 | 110154688 | rs17024393 | T | SNP | -0.9468 | -2.16 | 0.26 | 0.12 |
|  |  |  |  | Age | 0.0586 | 0.04 | 0.08 | 1.68E-10 |
|  |  |  |  | Sex | -0.5568 | -0.84 | -0.28 | 1.04E-04 |
|  |  |  |  | Income | -1.1600 | -2.51 | 0.19 | 0.0916 |
|  |  |  |  | SNPxIncome | 0.3022 | -0.39 | 1.00 | 0.39 |
| 1 | 177889480 | rs543874 | A | SNP | -0.1268 | -0.73 | 0.48 | 0.68 |
|  |  |  |  | Age | 0.0586 | 0.04 | 0.08 | 1.69E-10 |
|  |  |  |  | Sex | -0.5566 | -0.84 | -0.28 | 1.06E-04 |
|  |  |  |  | Income | -0.6482 | -1.24 | -0.06 | 0.03 |
|  |  |  |  | SNPxIncome | 0.0415 | -0.31 | 0.39 | 0.81 |
| 1 | 201784287 | rs2820292 | A | SNP | 0.0997 | -0.38 | 0.58 | 0.68 |
|  |  |  |  | Age | 0.0586 | 0.04 | 0.08 | 1.68E-10 |
|  |  |  |  | Sex | -0.5583 | -0.84 | -0.28 | 1.01E-04 |
|  |  |  |  | Income | -0.5526 | -0.88 | -0.23 | 8.03E-04 |
|  |  |  |  | SNPxIncome | -0.0330 | -0.31 | 0.25 | 0.82 |
| 2 | 632348 | rs13021737 | A | SNP | 0.0687 | -1.13 | 0.16 | 0.14 |
|  |  |  |  | Age | -0.8256 | 0.04 | 0.08 | 1.58E-10 |
|  |  |  |  | Sex | 0.2291 | -0.84 | -0.28 | 9.79E-05 |
|  |  |  |  | Income | -0.6045 | -0.84 | -0.37 | 6.19E-07 |
|  |  |  |  | SNPxIncome | 0.0687 | -0.31 | 0.44 | 0.72 |
| 2 | 25150296 | rs10182181 | A | SNP | -0.6718 | -1.05 | -0.1 | 0.02 |
|  |  |  |  | Age | 0.0595 | 0.04 | 0.08 | 1.38E-10 |
|  |  |  |  | Sex | -0.5604 | -0.84 | -0.28 | 1.00E-04 |
|  |  |  |  | Income | -0.8256 | -1.18 | -0.47 | 4.83E-06 |
|  |  |  |  | SNPxIncome | 0.2291 | -0.05 | 0.50 | 0.10 |
| 2 | 26928811 | rs11126666 | G | SNP | 0.0866 | -0.67 | 0.42 | 0.65 |
|  |  |  |  | Age | -0.2337 | 0.04 | 0.08 | 2.17E-10 |
|  |  |  |  | Sex | -0.2071 | -0.84 | -0.27 | 1.08E-04 |
|  |  |  |  | Income | -0.4777 | -0.98 | 0.02 | 0.06 |
|  |  |  |  | SNPxIncome | -0.0723 | -0.39 | 0.24 | 0.65 |
| 2 | 59305625 | rs1016287 | C | SNP | -0.3737 | -0.9 | 0.18 | 0.20 |
|  |  |  |  | Age | -0.1264 | 0.04 | 0.08 | 1.46E-10 |
|  |  |  |  | Sex | -0.7172 | -0.84 | -0.28 | 9.04E-05 |
|  |  |  |  | Income | -0.6718 | -1.16 | -0.18 | 0.01 |
|  |  |  |  | SNPxIncome | 0.0595 | -0.25 | 0.37 | 0.71 |
| 2 | 63053048 | rs11688816 | A | SNP | 0.1308 | -0.61 | 0.35 | 0.59 |
|  |  |  |  | Age | -1.0720 | 0.04 | 0.08 | 1.67E-10 |
|  |  |  |  | Sex | 0.3821 | -0.84 | -0.28 | 8.58E-05 |
|  |  |  |  | Income | -0.5604 | -0.88 | -0.24 | 0.001 |
|  |  |  |  | SNPxIncome | -0.0249 | -0.30 | 0.25 | 0.86 |
| 2 | 143043285 | rs2121279 | C | SNP | -0.5501 | -0.98 | 0.41 | 0.42 |
|  |  |  |  | Age | -0.0374 | 0.04 | 0.08 | 1.26E-10 |
|  |  |  |  | Sex | -1.0750 | -0.84 | -0.28 | 9.90E-05 |
|  |  |  |  | Income | -0.7293 | -1.47 | 0.01 | 0.05 |
|  |  |  |  | SNPxIncome | 0.0866 | -0.32 | 0.50 | 0.68 |
| 2 | 164567689 | rs1460676 | T | SNP | -0.3181 | -0.33 | 0.94 | 0.34 |
|  |  |  |  | Age | -0.5844 | 0.04 | 0.08 | 1.77E-10 |
|  |  |  |  | Sex | 0.0460 | -0.84 | -0.28 | 1.05E-04 |
|  |  |  |  | Income | -0.2337 | -0.89 | 0.42 | 0.48 |
|  |  |  |  | SNPxIncome | -0.2071 | -0.58 | 0.17 | 0.28 |
| 2 | 181550962 | rs1528435 | C | SNP | -0.4197 | -0.88 | 0.12 | 0.13 |
|  |  |  |  | Age | -0.1917 | 0.04 | 0.08 | 1.81E-10 |
|  |  |  |  | Sex | -0.5706 | -0.84 | -0.27 | 1.08E-04 |
|  |  |  |  | Income | -0.6777 | -0.96 | -0.39 | 2.91E-06 |
|  |  |  |  | SNPxIncome | 0.1377 | -0.15 | 0.43 | 0.35 |
| 2 | 208255518 | rs17203016 | A | SNP | -0.1084 | -0.46 | 0.75 | 0.64 |
|  |  |  |  | Age | -0.2283 | 0.04 | 0.08 | 2.10E-10 |
|  |  |  |  | Sex | -0.1954 | -0.84 | -0.28 | 8.99E-05 |
|  |  |  |  | Income | -0.3737 | -0.98 | 0.23 | 0.22 |
|  |  |  |  | SNPxIncome | -0.1264 | -0.48 | 0.23 | 0.48 |
| 2 | 213413231 | rs7599312 | A | SNP | -0.4217 | -0.74 | 0.33 | 0.44 |
|  |  |  |  | Age | -0.1900 | 0.04 | 0.08 | 1.85E-10 |
|  |  |  |  | Sex | -0.3180 | -0.85 | -0.28 | 8.37E-05 |
|  |  |  |  | Income | -0.7172 | -0.98 | -0.46 | 8.24E-08 |
|  |  |  |  | SNPxIncome | 0.2507 | -0.06 | 0.56 | 0.12 |
| 2 | 219349752 | rs492400 | T | SNP | 0.0301 | -0.73 | 0.26 | 0.35 |
|  |  |  |  | Age | -0.5449 | 0.04 | 0.08 | 1.99E-10 |
|  |  |  |  | Sex | -0.0828 | -0.84 | -0.28 | 1.04E-04 |
|  |  |  |  | Income | -0.7388 | -1.13 | -0.35 | 2.37E-04 |
|  |  |  |  | SNPxIncome | 0.1308 | -0.16 | 0.42 | 0.38 |
| 2 | 227092802 | rs2176040 | G | SNP | -0.5676 | -1.11 | -0.13 | 0.01 |
|  |  |  |  | Age | -0.0355 | 0.04 | 0.08 | 1.58E-10 |
|  |  |  |  | Sex | -0.4705 | -0.83 | -0.27 | 1.23E-04 |
|  |  |  |  | Income | -1.0720 | -1.48 | -0.66 | 3.56E-07 |
|  |  |  |  | SNPxIncome | 0.3821 | 0.10 | 0.66 | 0.01 |
| 3 | 25106437 | rs6804842 | A | SNP | -0.0878 | -0.76 | 0.2 | 0.26 |
|  |  |  |  | Age | -0.3798 | 0.04 | 0.08 | 1.85E-10 |
|  |  |  |  | Sex | -0.1756 | -0.84 | -0.28 | 1.05E-04 |
|  |  |  |  | Income | -0.6754 | -0.98 | -0.37 | 1.18E-05 |
|  |  |  |  | SNPxIncome | 0.1153 | -0.16 | 0.39 | 0.41 |
| 3 | 61236462 | rs2365389 | T | SNP | -0.5536 | -0.36 | 0.62 | 0.61 |
|  |  |  |  | Age | -0.0419 | 0.04 | 0.08 | 1.76E-10 |
|  |  |  |  | Sex | -0.6453 | -0.84 | -0.28 | 1.02E-04 |
|  |  |  |  | Income | -0.5501 | -0.86 | -0.24 | 5.66E-04 |
|  |  |  |  | SNPxIncome | -0.0374 | -0.33 | 0.25 | 0.80 |
| 3 | 81792112 | rs3849570 | C | SNP | 0.0463 | -1.24 | -0.23 | 0.00 |
|  |  |  |  | Age | -0.5051 | 0.04 | 0.08 | 2.37E-10 |
|  |  |  |  | Sex | -0.1505 | -0.84 | -0.28 | 8.37E-05 |
|  |  |  |  | Income | -1.0750 | -1.52 | -0.63 | 1.99E-06 |
|  |  |  |  | SNPxIncome | 0.3684 | 0.07 | 0.66 | 0.01 |
| 3 | 85807590 | rs13078960 | T | SNP | -0.5384 | -0.08 | 1.11 | 0.09 |
|  |  |  |  | Age | -0.0690 | 0.04 | 0.08 | 2.52E-10 |
|  |  |  |  | Sex | -0.5160 | -0.84 | -0.28 | 8.86E-05 |
|  |  |  |  | Income | -0.0797 | -0.66 | 0.50 | 0.79 |
|  |  |  |  | SNPxIncome | -0.3181 | -0.66 | 0.03 | 0.07 |
| 3 | 141306013 | rs2035935 **†** | G | SNP | 0.2011 | -0.88 | 1.03 | 0.87 |
|  |  |  |  | Age | -0.7736 | 0.04 | 0.08 | 1.42E-10 |
|  |  |  |  | Sex | 0.1931 | -0.84 | -0.28 | 9.63E-05 |
|  |  |  |  | Income | -0.5844 | -0.80 | -0.37 | 6.51E-08 |
|  |  |  |  | SNPxIncome | 0.0460 | -0.53 | 0.62 | 0.88 |
| 3 | 185824004 | rs1516725 | T | SNP | -0.6171 | -0.29 | 1.13 | 0.24 |
|  |  |  |  | Age | 0.0228 | 0.04 | 0.08 | 1.65E-10 |
|  |  |  |  | Sex | -0.5563 | -0.84 | -0.27 | 1.10E-04 |
|  |  |  |  | Income | -0.5471 | -0.77 | -0.32 | 2.11E-06 |
|  |  |  |  | SNPxIncome | -0.1322 | -0.54 | 0.28 | 0.53 |
| 4 | 45182527 | rs10938397 | G | SNP | 0.2603 | 0.06 | 1.04 | 0.03 |
|  |  |  |  | Age | -0.5447 | 0.04 | 0.08 | 1.70E-10 |
|  |  |  |  | Sex | -0.0492 | -0.83 | -0.27 | 1.15E-04 |
|  |  |  |  | Income | -0.4197 | -0.73 | -0.11 | 0.01 |
|  |  |  |  | SNPxIncome | -0.1917 | -0.48 | 0.09 | 0.18 |
| 4 | 77096118 | rs17001561 **†** | A | SNP | -0.4590 | -0.76 | 0.59 | 0.81 |
|  |  |  |  | Age | -0.2604 | 0.04 | 0.08 | 1.52E-10 |
|  |  |  |  | Sex | -0.6608 | -0.84 | -0.28 | 1.02E-04 |
|  |  |  |  | Income | -0.5706 | -0.80 | -0.34 | 1.50E-06 |
|  |  |  |  | SNPxIncome | -0.0284 | -0.42 | 0.36 | 0.89 |
| 4 | 103188709 | rs13107325 | T | SNP | 0.0475 | -0.52 | 1.47 | 0.35 |
|  |  |  |  | Age | -0.6716 | 0.04 | 0.08 | 1.42E-10 |
|  |  |  |  | Sex | 0.0749 | -0.84 | -0.28 | 1.03E-04 |
|  |  |  |  | Income | -0.5680 | -0.78 | -0.36 | 1.47E-07 |
|  |  |  |  | SNPxIncome | -0.1084 | -0.69 | 0.47 | 0.71 |
| 4 | 145659064 | rs11727676 | T | SNP | -1.0970 | -0.45 | 1.18 | 0.38 |
|  |  |  |  | Age | 0.2918 | 0.04 | 0.08 | 2.13E-10 |
|  |  |  |  | Sex | -0.3184 | -0.84 | -0.28 | 9.06E-05 |
|  |  |  |  | Income | -0.2283 | -1.12 | 0.66 | 0.61 |
|  |  |  |  | SNPxIncome | -0.1954 | -0.67 | 0.28 | 0.42 |
| 5 | 75015242 | rs2112347 | G | SNP | -0.0140 | -0.6 | 0.41 | 0.73 |
|  |  |  |  | Age | -0.4264 | 0.04 | 0.08 | 1.54E-10 |
|  |  |  |  | Sex | -0.2630 | -0.84 | -0.28 | 1.01E-04 |
|  |  |  |  | Income | -0.6049 | -0.89 | -0.32 | 4.31E-05 |
|  |  |  |  | SNPxIncome | 0.0328 | -0.26 | 0.32 | 0.83 |
| 5 | 153537893 | rs7715256 | G | SNP | -0.6023 | -0.11 | 0.87 | 0.13 |
|  |  |  |  | Age | 0.2290 | 0.04 | 0.08 | 1.68E-10 |
|  |  |  |  | Sex | -0.8152 | -0.84 | -0.27 | 1.12E-04 |
|  |  |  |  | Income | -0.4217 | -0.73 | -0.11 | 0.01 |
|  |  |  |  | SNPxIncome | -0.1900 | -0.47 | 0.09 | 0.19 |
| 6 | 34563164 | rs205262 | A | SNP | 0.1530 | -0.49 | 0.57 | 0.89 |
|  |  |  |  | Age | -0.6542 | 0.04 | 0.08 | 2.07E-10 |
|  |  |  |  | Sex | 0.1547 | -0.84 | -0.28 | 8.56E-05 |
|  |  |  |  | Income | -0.3180 | -0.80 | 0.16 | 0.20 |
|  |  |  |  | SNPxIncome | -0.1841 | -0.49 | 0.12 | 0.24 |
| 6 | 40348653 | rs2033529 | G | SNP | -0.4699 | -0.57 | 0.47 | 0.86 |
|  |  |  |  | Age | -0.2269 | 0.04 | 0.08 | 1.60E-10 |
|  |  |  |  | Sex | -0.5676 | -0.84 | -0.28 | 1.03E-04 |
|  |  |  |  | Income | -0.5988 | -0.87 | -0.33 | 1.22E-05 |
|  |  |  |  | SNPxIncome | 0.0301 | -0.27 | 0.33 | 0.85 |
| 6 | 50845490 | rs2207139 | G | SNP | -0.1481 | 0 | 1.21 | 0.05 |
|  |  |  |  | Age | -0.4878 | 0.04 | 0.08 | 5.71E-11 |
|  |  |  |  | Sex | -0.1188 | -0.83 | -0.27 | 1.14E-04 |
|  |  |  |  | Income | -0.5449 | -0.78 | -0.31 | 6.25E-06 |
|  |  |  |  | SNPxIncome | -0.0828 | -0.43 | 0.27 | 0.64 |
| 6 | 108977663 | rs9400239 | T | SNP | -0.8101 | -0.93 | 0.1 | 0.11 |
|  |  |  |  | Age | 0.2997 | 0.04 | 0.08 | 1.84E-10 |
|  |  |  |  | Sex | -0.6763 | -0.85 | -0.29 | 7.24E-05 |
|  |  |  |  | Income | -0.6566 | -0.93 | -0.39 | 1.77E-06 |
|  |  |  |  | SNPxIncome | 0.1151 | -0.18 | 0.41 | 0.44 |
| 6 | 120185665 | rs9374842 | C | SNP | -0.1038 | -0.58 | 0.55 | 0.96 |
|  |  |  |  | Age | -0.5694 | 0.04 | 0.08 | 1.61E-10 |
|  |  |  |  | Sex | -0.0318 | -0.84 | -0.28 | 8.76E-05 |
|  |  |  |  | Income | -0.5676 | -0.82 | -0.31 | 1.44E-05 |
|  |  |  |  | SNPxIncome | -0.0355 | -0.37 | 0.30 | 0.83 |
| 6 | 137675541 | rs13201877 | A | SNP | -0.6306 | -0.6 | 0.71 | 0.88 |
|  |  |  |  | Age | 0.0571 | 0.04 | 0.08 | 1.58E-10 |
|  |  |  |  | Sex | -0.4044 | -0.84 | -0.28 | 9.59E-05 |
|  |  |  |  | Income | -0.4705 | -1.12 | 0.18 | 0.16 |
|  |  |  |  | SNPxIncome | -0.0665 | -0.44 | 0.31 | 0.73 |
| 6 | 163033350 | rs13191362 | G | SNP | -0.1609 | -0.61 | 1 | 0.64 |
|  |  |  |  | Age | -0.4910 | 0.04 | 0.08 | 1.88E-10 |
|  |  |  |  | Sex | -0.2806 | -0.83 | -0.27 | 1.19E-04 |
|  |  |  |  | Income | -0.5652 | -0.79 | -0.34 | 5.79E-07 |
|  |  |  |  | SNPxIncome | -0.0878 | -0.56 | 0.38 | 0.71 |
| 7 | 75163169 | rs1167827 | G | SNP | -0.5158 | -0.11 | 0.85 | 0.13 |
|  |  |  |  | Age | -0.1263 | 0.04 | 0.08 | 1.79E-10 |
|  |  |  |  | Sex | -0.4031 | -0.84 | -0.28 | 9.03E-05 |
|  |  |  |  | Income | -0.3798 | -0.76 | 0.00 | 0.05 |
|  |  |  |  | SNPxIncome | -0.1756 | -0.46 | 0.10 | 0.22 |
| 7 | 76608143 | rs2245368 | C | SNP | -0.1175 | -0.67 | 0.63 | 0.96 |
|  |  |  |  | Age | -0.5116 | 0.04 | 0.08 | 2.75E-10 |
|  |  |  |  | Sex | -0.1523 | -0.84 | -0.28 | 9.97E-05 |
|  |  |  |  | Income | -0.6071 | -0.84 | -0.37 | 4.81E-07 |
|  |  |  |  | SNPxIncome | 0.0625 | -0.32 | 0.45 | 0.75 |
| 7 | 93568420 | rs9641123 | C | SNP | 0.0062 | -0.53 | 0.54 | 0.98 |
|  |  |  |  | Age | 0.0597 | 0.04 | 0.08 | 1.42E-09 |
|  |  |  |  | Sex | -0.7036 | -1.01 | -0.40 | 5.09E-06 |
|  |  |  |  | Income | -0.6242 | -0.95 | -0.30 | 1.82E-04 |
|  |  |  |  | SNPxIncome | 0.0146 | -0.29 | 0.32 | 0.93 |
| 7 | 95169514 | rs6465468 | T | SNP | -0.6084 | -0.57 | 0.45 | 0.81 |
|  |  |  |  | Age | 0.0561 | 0.04 | 0.08 | 1.67E-10 |
|  |  |  |  | Sex | -0.6156 | -0.84 | -0.28 | 9.23E-05 |
|  |  |  |  | Income | -0.5536 | -0.82 | -0.28 | 6.17E-05 |
|  |  |  |  | SNPxIncome | -0.0419 | -0.34 | 0.25 | 0.78 |
| 8 | 76806584 | rs17405819 | T | SNP | 0.1375 | -0.39 | 0.66 | 0.61 |
|  |  |  |  | Age | 0.0585 | 0.04 | 0.08 | 1.78E-10 |
|  |  |  |  | Sex | -0.5625 | -0.84 | -0.28 | 8.89E-05 |
|  |  |  |  | Income | -0.6453 | -1.12 | -0.17 | 0.01 |
|  |  |  |  | SNPxIncome | 0.0433 | -0.26 | 0.35 | 0.78 |
| 8 | 81375457 | rs16907751 | C | SNP | -0.0032 | -0.8 | 0.79 | 0.99 |
|  |  |  |  | Age | 0.0586 | 0.04 | 0.08 | 1.72E-10 |
|  |  |  |  | Sex | -0.5597 | -0.84 | -0.28 | 9.66E-05 |
|  |  |  |  | Income | -0.6626 | -1.50 | 0.17 | 0.1205 |
|  |  |  |  | SNPxIncome | 0.0463 | -0.41 | 0.50 | 0.84 |
| 8 | 85079709 | rs2033732 | T | SNP | 0.2982 | -0.24 | 0.84 | 0.28 |
|  |  |  |  | Age | 0.0585 | 0.04 | 0.08 | 1.72E-10 |
|  |  |  |  | Sex | -0.5574 | -0.84 | -0.28 | 1.03E-04 |
|  |  |  |  | Income | -0.5051 | -0.76 | -0.25 | 1.03E-04 |
|  |  |  |  | SNPxIncome | -0.1505 | -0.47 | 0.17 | 0.35 |
| 9 | 15634326 | rs4740619 | T | SNP | 0.0000 | -0.49 | 0.49 | 1.00 |
|  |  |  |  | Age | 0.0587 | 0.04 | 0.08 | 1.60E-10 |
|  |  |  |  | Sex | -0.5585 | -0.84 | -0.28 | 9.97E-05 |
|  |  |  |  | Income | -0.5914 | -0.97 | -0.21 | 2.28E-03 |
|  |  |  |  | SNPxIncome | 0.0089 | -0.28 | 0.29 | 0.95 |
| 9 | 28414339 | rs10968576 | G | SNP | 0.1706 | -0.36 | 0.7 | 0.53 |
|  |  |  |  | Age | 0.0589 | 0.04 | 0.08 | 1.37E-10 |
|  |  |  |  | Sex | -0.5573 | -0.84 | -0.28 | 1.03E-04 |
|  |  |  |  | Income | -0.5384 | -0.81 | -0.27 | 1.16E-04 |
|  |  |  |  | SNPxIncome | -0.0690 | -0.38 | 0.24 | 0.67 |
| 9 | 111932342 | rs6477694 | C | SNP | 0.1414 | -0.35 | 0.64 | 0.58 |
|  |  |  |  | Age | 0.0585 | 0.04 | 0.08 | 1.75E-10 |
|  |  |  |  | Sex | -0.5561 | -0.84 | -0.28 | 1.07E-04 |
|  |  |  |  | Income | -0.5160 | -0.81 | -0.23 | 5.16E-04 |
|  |  |  |  | SNPxIncome | -0.0886 | -0.38 | 0.20 | 0.55 |
| 9 | 120378483 | rs1928295 | C | SNP | -0.4103 | -0.9 | 0.08 | 0.10 |
|  |  |  |  | Age | 0.0585 | 0.04 | 0.08 | 1.73E-10 |
|  |  |  |  | Sex | -0.5510 | -0.83 | -0.27 | 1.24E-04 |
|  |  |  |  | Income | -0.7712 | -1.10 | -0.44 | 5.46E-06 |
|  |  |  |  | SNPxIncome | 0.2011 | -0.08 | 0.48 | 0.16 |
| 9 | 129460914 | rs10733682 | A | SNP | -0.4466 | -0.93 | 0.04 | 0.07 |
|  |  |  |  | Age | 0.0582 | 0.04 | 0.08 | 2.13E-10 |
|  |  |  |  | Sex | -0.5692 | -0.85 | -0.29 | 7.37E-05 |
|  |  |  |  | Income | -0.7736 | -1.12 | -0.43 | 1.00E-05 |
|  |  |  |  | SNPxIncome | 0.1931 | -0.09 | 0.47 | 0.18 |
| 10 | 87410904 | rs7899106 | G | SNP | 0.1223 | -1.02 | 1.27 | 0.83 |
|  |  |  |  | Age | 0.0586 | 0.04 | 0.08 | 1.62E-10 |
|  |  |  |  | Sex | -0.5607 | -0.84 | -0.28 | 9.44E-05 |
|  |  |  |  | Income | -0.5914 | -0.80 | -0.38 | 3.36E-08 |
|  |  |  |  | SNPxIncome | 0.1094 | -0.56 | 0.78 | 0.75 |
| 10 | 102395440 | rs17094222 | T | SNP | -0.1399 | -0.74 | 0.46 | 0.65 |
|  |  |  |  | Age | 0.0588 | 0.04 | 0.08 | 1.46E-10 |
|  |  |  |  | Sex | -0.5585 | -0.84 | -0.28 | 1.00E-04 |
|  |  |  |  | Income | -0.6171 | -1.20 | -0.03 | 0.04 |
|  |  |  |  | SNPxIncome | 0.0228 | -0.32 | 0.37 | 0.90 |
| 10 | 104869038 | rs11191560 | C | SNP | 0.3331 | -0.47 | 1.14 | 0.42 |
|  |  |  |  | Age | 0.0587 | 0.04 | 0.08 | 1.56E-10 |
|  |  |  |  | Sex | -0.5602 | -0.84 | -0.28 | 9.51E-05 |
|  |  |  |  | Income | -0.5563 | -0.78 | -0.34 | 7.09E-07 |
|  |  |  |  | SNPxIncome | -0.1285 | -0.59 | 0.33 | 0.58 |
| 10 | 114758349 | rs7903146 | T | SNP | -0.5769 | -1.11 | -0.04 | 0.03 |
|  |  |  |  | Age | 0.0587 | 0.04 | 0.08 | 1.48E-10 |
|  |  |  |  | Sex | -0.5652 | -0.85 | -0.28 | 8.17E-05 |
|  |  |  |  | Income | -0.7244 | -0.99 | -0.46 | 6.72E-08 |
|  |  |  |  | SNPxIncome | 0.2603 | -0.05 | 0.57 | 0.10 |
| 11 | 8673939 | rs4256980 | C | SNP | -0.0063 | -0.51 | 0.49 | 0.98 |
|  |  |  |  | Age | 0.0587 | 0.04 | 0.08 | 1.56E-10 |
|  |  |  |  | Sex | -0.5573 | -0.84 | -0.28 | 1.03E-04 |
|  |  |  |  | Income | -0.5447 | -0.84 | -0.25 | 2.69E-04 |
|  |  |  |  | SNPxIncome | -0.0492 | -0.34 | 0.24 | 0.74 |
| 11 | 27684517 | rs11030104 | G | SNP | -0.7258 | -1.32 | -0.14 | 0.02 |
|  |  |  |  | Age | 0.0589 | 0.04 | 0.08 | 1.23E-10 |
|  |  |  |  | Sex | -0.5610 | -0.84 | -0.28 | 9.09E-05 |
|  |  |  |  | Income | -0.6724 | -0.92 | -0.43 | 1.00E-07 |
|  |  |  |  | SNPxIncome | 0.2115 | -0.13 | 0.55 | 0.22 |
| 11 | 43864278 | rs2176598 | T | SNP | 0.5231 | -0.03 | 1.07 | 0.06 |
|  |  |  |  | Age | 0.0583 | 0.04 | 0.08 | 2.05E-10 |
|  |  |  |  | Sex | -0.5688 | -0.85 | -0.29 | 7.45E-05 |
|  |  |  |  | Income | -0.4590 | -0.71 | -0.21 | 3.46E-04 |
|  |  |  |  | SNPxIncome | -0.2604 | -0.58 | 0.06 | 0.11 |
| 11 | 47650993 | rs3817334 | T | SNP | -0.1042 | -0.59 | 0.38 | 0.67 |
|  |  |  |  | Age | 0.0587 | 0.04 | 0.08 | 1.55E-10 |
|  |  |  |  | Sex | -0.5550 | -0.84 | -0.27 | 1.11E-04 |
|  |  |  |  | Income | -0.6608 | -0.97 | -0.35 | 2.49E-05 |
|  |  |  |  | SNPxIncome | 0.0953 | -0.18 | 0.37 | 0.50 |
| 11 | 115022404 | rs12286929 | A | SNP | -0.3009 | -0.79 | 0.18 | 0.22 |
|  |  |  |  | Age | 0.0586 | 0.04 | 0.08 | 1.56E-10 |
|  |  |  |  | Sex | -0.5598 | -0.84 | -0.28 | 9.51E-05 |
|  |  |  |  | Income | -0.6230 | -0.95 | -0.30 | 1.54E-04 |
|  |  |  |  | SNPxIncome | 0.0475 | -0.23 | 0.33 | 0.74 |
| 12 | 50247468 | rs7138803 | G | SNP | -0.3289 | -0.83 | 0.17 | 0.20 |
|  |  |  |  | Age | 0.0588 | 0.04 | 0.08 | 1.38E-10 |
|  |  |  |  | Sex | -0.5554 | -0.84 | -0.27 | 1.08E-04 |
|  |  |  |  | Income | -0.6716 | -1.08 | -0.27 | 0.001 |
|  |  |  |  | SNPxIncome | 0.0749 | -0.22 | 0.36 | 0.61 |
| 12 | 122781897 | rs11057405 | G | SNP | 0.3332 | -0.44 | 1.1 | 0.40 |
|  |  |  |  | Age | 0.0584 | 0.04 | 0.08 | 1.87E-10 |
|  |  |  |  | Sex | -0.5592 | -0.84 | -0.28 | 9.78E-05 |
|  |  |  |  | Income | -0.5188 | -1.31 | 0.27 | 0.20 |
|  |  |  |  | SNPxIncome | -0.0310 | -0.46 | 0.40 | 0.89 |
| 13 | 27443877 | rs9581855 **†** | C | SNP | 0.6589 | 0.04 | 1.28 | 0.04 |
|  |  |  |  | Age | 0.0595 | 0.04 | 0.08 | 1.54E-09 |
|  |  |  |  | Sex | -0.7033 | -1.00 | -0.40 | 5.01E-06 |
|  |  |  |  | Income | -0.5233 | -0.79 | -0.26 | 9.75E-05 |
|  |  |  |  | SNPxIncome | -0.2050 | -0.57 | 0.16 | 0.27 |
| 13 | 54102206 | rs12429545 | G | SNP | -0.6772 | -1.43 | 0.08 | 0.08 |
|  |  |  |  | Age | 0.0584 | 0.04 | 0.08 | 1.95E-10 |
|  |  |  |  | Sex | -0.5656 | -0.85 | -0.28 | 8.13E-05 |
|  |  |  |  | Income | -1.0970 | -1.87 | -0.33 | 0.005 |
|  |  |  |  | SNPxIncome | 0.2918 | -0.13 | 0.72 | 0.18 |
| 13 | 66205704 | rs9540493 | A | SNP | 0.7136 | 0.24 | 1.19 | 0.003 |
|  |  |  |  | Age | 0.0590 | 0.04 | 0.08 | 1.19E-10 |
|  |  |  |  | Sex | -0.5595 | -0.84 | -0.28 | 9.54E-05 |
|  |  |  |  | Income | -0.3184 | -0.62 | -0.01 | 0.04 |
|  |  |  |  | SNPxIncome | -0.3124 | -0.59 | -0.04 | 0.02 |
| 13 | 79580919 | rs1441264 | A | SNP | 0.1157 | -0.38 | 0.61 | 0.65 |
|  |  |  |  | Age | 0.0587 | 0.04 | 0.08 | 1.55E-10 |
|  |  |  |  | Sex | -0.5579 | -0.84 | -0.28 | 1.02E-04 |
|  |  |  |  | Income | -0.5647 | -0.96 | -0.17 | 0.005 |
|  |  |  |  | SNPxIncome | -0.0140 | -0.30 | 0.27 | 0.92 |
| 14 | 25928179 | rs10132280 | A | SNP | 0.4415 | -0.09 | 0.97 | 0.10 |
|  |  |  |  | Age | 0.0587 | 0.04 | 0.08 | 1.50E-10 |
|  |  |  |  | Sex | -0.5553 | -0.84 | -0.27 | 1.09E-04 |
|  |  |  |  | Income | -0.4264 | -0.70 | -0.16 | 0.002 |
|  |  |  |  | SNPxIncome | -0.2630 | -0.57 | 0.05 | 0.10 |
| 14 | 29736838 | rs12885454 | C | SNP | 0.3160 | -0.18 | 0.81 | 0.21 |
|  |  |  |  | Age | 0.0586 | 0.04 | 0.08 | 1.64E-10 |
|  |  |  |  | Sex | -0.5616 | -0.84 | -0.28 | 9.10E-05 |
|  |  |  |  | Income | -0.4399 | -0.86 | -0.02 | 0.04 |
|  |  |  |  | SNPxIncome | -0.1089 | -0.40 | 0.18 | 0.46 |
| 14 | 30515112 | rs11847697 | T | SNP | -0.0254 | -1.18 | 1.13 | 0.97 |
|  |  |  |  | Age | 0.0587 | 0.04 | 0.08 | 1.52E-10 |
|  |  |  |  | Sex | -0.5574 | -0.84 | -0.28 | 1.03E-04 |
|  |  |  |  | Income | -0.6023 | -0.81 | -0.39 | 1.71E-08 |
|  |  |  |  | SNPxIncome | 0.2290 | -0.44 | 0.90 | 0.50 |
| 14 | 79899454 | rs7141420 | C | SNP | -0.2837 | -0.77 | 0.21 | 0.26 |
|  |  |  |  | Age | 0.0586 | 0.04 | 0.08 | 1.69E-10 |
|  |  |  |  | Sex | -0.5614 | -0.84 | -0.28 | 9.10E-05 |
|  |  |  |  | Income | -0.8152 | -1.15 | -0.48 | 2.16E-06 |
|  |  |  |  | SNPxIncome | 0.2460 | -0.04 | 0.53 | 0.09 |
| 15 | 51748610 | rs3736485 | A | SNP | -0.2191 | -0.7 | 0.26 | 0.37 |
|  |  |  |  | Age | 0.0585 | 0.04 | 0.08 | 1.73E-10 |
|  |  |  |  | Sex | -0.5606 | -0.84 | -0.28 | 9.37E-05 |
|  |  |  |  | Income | -0.7132 | -1.03 | -0.40 | 8.30E-06 |
|  |  |  |  | SNPxIncome | 0.1530 | -0.13 | 0.43 | 0.28 |
| 15 | 68077168 | rs16951275 | C | SNP | -0.3709 | -0.94 | 0.2 | 0.20 |
|  |  |  |  | Age | 0.0585 | 0.04 | 0.08 | 1.71E-10 |
|  |  |  |  | Sex | -0.5612 | -0.84 | -0.28 | 9.21E-05 |
|  |  |  |  | Income | -0.6542 | -0.91 | -0.40 | 5.83E-07 |
|  |  |  |  | SNPxIncome | 0.1547 | -0.18 | 0.48 | 0.36 |
| 15 | 73093991 | rs7164727 | C | SNP | -0.2604 | -0.77 | 0.25 | 0.32 |
|  |  |  |  | Age | 0.0589 | 0.04 | 0.08 | 1.35E-10 |
|  |  |  |  | Sex | -0.5605 | -0.84 | -0.28 | 9.40E-05 |
|  |  |  |  | Income | -0.6342 | -0.91 | -0.36 | 6.37E-06 |
|  |  |  |  | SNPxIncome | 0.0835 | -0.22 | 0.38 | 0.59 |
| 16 | 3627358 | rs758747 | T | SNP | 0.4135 | -0.15 | 0.97 | 0.15 |
|  |  |  |  | Age | 0.0587 | 0.04 | 0.08 | 1.58E-10 |
|  |  |  |  | Sex | -0.5629 | -0.84 | -0.28 | 8.78E-05 |
|  |  |  |  | Income | -0.4699 | -0.73 | -0.21 | 3.25E-04 |
|  |  |  |  | SNPxIncome | -0.2269 | -0.56 | 0.10 | 0.18 |
| 16 | 19935389 | rs12446632 | A | SNP | 0.0530 | -0.62 | 0.73 | 0.88 |
|  |  |  |  | Age | 0.0589 | 0.04 | 0.08 | 1.41E-10 |
|  |  |  |  | Sex | -0.5668 | -0.85 | -0.29 | 7.91E-05 |
|  |  |  |  | Income | -0.5676 | -0.80 | -0.34 | 1.40E-06 |
|  |  |  |  | SNPxIncome | -0.0560 | -0.44 | 0.33 | 0.77 |
| 16 | 28333411 | rs2650492 | A | SNP | 0.1392 | -0.38 | 0.65 | 0.60 |
|  |  |  |  | Age | 0.0586 | 0.04 | 0.08 | 1.68E-10 |
|  |  |  |  | Sex | -0.5577 | -0.84 | -0.28 | 1.02E-04 |
|  |  |  |  | Income | -0.4844 | -0.76 | -0.21 | 6.42E-04 |
|  |  |  |  | SNPxIncome | -0.1481 | -0.45 | 0.15 | 0.33 |
| 16 | 28889486 | rs3888190 | A | SNP | 0.1542 | -0.34 | 0.65 | 0.54 |
|  |  |  |  | Age | 0.0586 | 0.04 | 0.08 | 1.61E-10 |
|  |  |  |  | Sex | -0.5600 | -0.84 | -0.28 | 9.54E-05 |
|  |  |  |  | Income | -0.4878 | -0.79 | -0.19 | 0.001 |
|  |  |  |  | SNPxIncome | -0.1188 | -0.40 | 0.17 | 0.41 |
| 16 | 30015337 | rs4787491 | A | SNP | -0.3379 | -0.81 | 0.13 | 0.16 |
|  |  |  |  | Age | 0.0584 | 0.04 | 0.08 | 1.92E-10 |
|  |  |  |  | Sex | -0.5540 | -0.84 | -0.27 | 1.13E-04 |
|  |  |  |  | Income | -0.7802 | -1.11 | -0.45 | 3.54E-06 |
|  |  |  |  | SNPxIncome | 0.2076 | -0.07 | 0.48 | 0.14 |
| 16 | 31129895 | rs9925964 | G | SNP | -0.5617 | -1.07 | -0.06 | 0.03 |
|  |  |  |  | Age | 0.0590 | 0.04 | 0.08 | 1.21E-10 |
|  |  |  |  | Sex | -0.5632 | -0.84 | -0.28 | 8.65E-05 |
|  |  |  |  | Income | -0.8101 | -1.11 | -0.51 | 1.33E-07 |
|  |  |  |  | SNPxIncome | 0.2997 | 0.01 | 0.59 | 0.04 |
| 16 | 49062590 | rs2080454 | C | SNP | -0.0732 | -0.56 | 0.42 | 0.77 |
|  |  |  |  | Age | 0.0584 | 0.04 | 0.08 | 1.87E-10 |
|  |  |  |  | Sex | -0.5558 | -0.84 | -0.27 | 1.08E-04 |
|  |  |  |  | Income | -0.6763 | -0.97 | -0.38 | 8.83E-06 |
|  |  |  |  | SNPxIncome | 0.1221 | -0.16 | 0.41 | 0.40 |
| 16 | 53803574 | rs1558902 | A | SNP | 0.4428 | -0.05 | 0.93 | 0.08 |
|  |  |  |  | Age | 0.0581 | 0.04 | 0.08 | 2.40E-10 |
|  |  |  |  | Sex | -0.5533 | -0.83 | -0.27 | 1.16E-04 |
|  |  |  |  | Income | -0.4908 | -0.81 | -0.17 | 0.003 |
|  |  |  |  | SNPxIncome | -0.1038 | -0.39 | 0.18 | 0.48 |
| 17 | 2005136 | rs9914578 | G | SNP | 0.0807 | -0.52 | 0.69 | 0.79 |
|  |  |  |  | Age | 0.0586 | 0.04 | 0.08 | 1.74E-10 |
|  |  |  |  | Sex | -0.5571 | -0.84 | -0.28 | 1.04E-04 |
|  |  |  |  | Income | -0.5694 | -0.81 | -0.33 | 4.42E-06 |
|  |  |  |  | SNPxIncome | -0.0318 | -0.38 | 0.32 | 0.86 |
| 17 | 5283252 | rs1000940 | G | SNP | 0.1171 | -0.42 | 0.65 | 0.67 |
|  |  |  |  | Age | 0.0591 | 0.04 | 0.08 | 1.15E-10 |
|  |  |  |  | Sex | -0.5571 | -0.84 | -0.28 | 1.03E-04 |
|  |  |  |  | Income | -0.6220 | -0.89 | -0.35 | 7.03E-06 |
|  |  |  |  | SNPxIncome | 0.0645 | -0.25 | 0.37 | 0.68 |
| 17 | 78615571 | rs12940622 | A | SNP | -0.1102 | -0.6 | 0.38 | 0.66 |
|  |  |  |  | Age | 0.0588 | 0.04 | 0.08 | 1.48E-10 |
|  |  |  |  | Sex | -0.5575 | -0.84 | -0.28 | 1.03E-04 |
|  |  |  |  | Income | -0.6306 | -0.95 | -0.31 | 1.03E-04 |
|  |  |  |  | SNPxIncome | 0.0571 | -0.22 | 0.34 | 0.69 |
| 18 | 21104888 | rs1808579 | T | SNP | 0.3155 | -0.16 | 0.79 | 0.19 |
|  |  |  |  | Age | 0.0586 | 0.04 | 0.08 | 1.58E-10 |
|  |  |  |  | Sex | -0.5514 | -0.83 | -0.27 | 1.22E-04 |
|  |  |  |  | Income | -0.4044 | -0.73 | -0.08 | 0.02 |
|  |  |  |  | SNPxIncome | -0.1907 | -0.47 | 0.09 | 0.18 |
| 18 | 40147671 | rs7239883 | G | SNP | 0.3934 | -0.1 | 0.88 | 0.12 |
|  |  |  |  | Age | 0.0583 | 0.04 | 0.08 | 2.06E-10 |
|  |  |  |  | Sex | -0.5609 | -0.84 | -0.28 | 9.33E-05 |
|  |  |  |  | Income | -0.4613 | -0.76 | -0.16 | 0.002 |
|  |  |  |  | SNPxIncome | -0.1609 | -0.45 | 0.12 | 0.27 |
| 18 | 56883319 | rs7243357 | G | SNP | 0.4736 | -0.19 | 1.14 | 0.16 |
|  |  |  |  | Age | 0.0583 | 0.04 | 0.08 | 2.12E-10 |
|  |  |  |  | Sex | -0.5540 | -0.84 | -0.27 | 1.14E-04 |
|  |  |  |  | Income | -0.4910 | -0.73 | -0.26 | 4.51E-05 |
|  |  |  |  | SNPxIncome | -0.2806 | -0.67 | 0.11 | 0.16 |
| 18 | 57829135 | rs6567160 | C | SNP | -0.2286 | -0.78 | 0.32 | 0.42 |
|  |  |  |  | Age | 0.0589 | 0.04 | 0.08 | 1.29E-10 |
|  |  |  |  | Sex | -0.5605 | -0.84 | -0.28 | 9.23E-05 |
|  |  |  |  | Income | -0.7427 | -1.00 | -0.49 | 1.04E-08 |
|  |  |  |  | SNPxIncome | 0.3063 | -0.01 | 0.62 | 0.06 |
| 19 | 18454825 | rs17724992 | G | SNP | 0.1272 | -0.42 | 0.67 | 0.65 |
|  |  |  |  | Age | 0.0585 | 0.04 | 0.08 | 1.79E-10 |
|  |  |  |  | Sex | -0.5589 | -0.84 | -0.28 | 9.85E-05 |
|  |  |  |  | Income | -0.5158 | -0.77 | -0.26 | 7.55E-05 |
|  |  |  |  | SNPxIncome | -0.1263 | -0.44 | 0.19 | 0.43 |
| 19 | 34309532 | rs29941 | A | SNP | 0.3917 | -0.12 | 0.91 | 0.14 |
|  |  |  |  | Age | 0.0585 | 0.04 | 0.08 | 1.78E-10 |
|  |  |  |  | Sex | -0.5594 | -0.84 | -0.28 | 9.69E-05 |
|  |  |  |  | Income | -0.4031 | -0.67 | -0.14 | 0.003 |
|  |  |  |  | SNPxIncome | -0.3010 | -0.60 | 0.00 | 0.05 |
| 19 | 45395619 | rs2075650 | G | SNP | 0.2685 | -0.41 | 0.95 | 0.44 |
|  |  |  |  | Age | 0.0587 | 0.04 | 0.08 | 1.58E-10 |
|  |  |  |  | Sex | -0.5562 | -0.84 | -0.28 | 1.07E-04 |
|  |  |  |  | Income | -0.5461 | -0.78 | -0.32 | 3.23E-06 |
|  |  |  |  | SNPxIncome | -0.1175 | -0.52 | 0.29 | 0.57 |
| 19 | 46202172 | rs2287019 | T | SNP | -0.0941 | -0.68 | 0.49 | 0.75 |
|  |  |  |  | Age | 0.0585 | 0.04 | 0.08 | 1.76E-10 |
|  |  |  |  | Sex | -0.5555 | -0.84 | -0.27 | 1.07E-04 |
|  |  |  |  | Income | -0.5116 | -0.76 | -0.26 | 5.07E-05 |
|  |  |  |  | SNPxIncome | -0.1523 | -0.49 | 0.18 | 0.37 |
| 19 | 47569003 | rs3810291 | G | SNP | -0.0962 | -0.61 | 0.41 | 0.71 |
|  |  |  |  | Age | 0.0583 | 0.04 | 0.08 | 2.07E-10 |
|  |  |  |  | Sex | -0.5597 | -0.84 | -0.28 | 9.71E-05 |
|  |  |  |  | Income | -0.5622 | -0.84 | -0.29 | 7.14E-05 |
|  |  |  |  | SNPxIncome | -0.0269 | -0.32 | 0.27 | 0.86 |
| 20 | 51087862 | rs6091540 | T | SNP | -0.1945 | -0.75 | 0.36 | 0.49 |
|  |  |  |  | Age | 0.0587 | 0.04 | 0.08 | 1.49E-10 |
|  |  |  |  | Sex | -0.5582 | -0.84 | -0.28 | 1.01E-04 |
|  |  |  |  | Income | -0.6084 | -0.87 | -0.35 | 4.93E-06 |
|  |  |  |  | SNPxIncome | 0.0561 | -0.26 | 0.38 | 0.73 |
| 21 | 40291740 | rs2836754 | T | SNP | -0.1462 | -0.64 | 0.35 | 0.56 |
|  |  |  |  | Age | 0.0587 | 0.04 | 0.08 | 1.58E-10 |
|  |  |  |  | Sex | -0.5599 | -0.84 | -0.28 | 9.58E-05 |
|  |  |  |  | Income | -0.6156 | -0.91 | -0.32 | 4.30E-05 |
|  |  |  |  | SNPxIncome | 0.0465 | -0.24 | 0.33 | 0.75 |

*Position of build 36; † proxy SNPs (rs2035935 as a proxy for rs16851483, rs17001561 as a proxy for rs17001654 and rs9581855 as a proxy for rs12016871), LD of proxy SNPs r²>0.9.
