# Supplementary material for: A genetic sum score of risk alleles associated with body mass index interacts with socioeconomic position in the Heinz Nixdorf Recall Study
Source: PLoS One. 2019 Aug 23;14(8):e0221252. doi: 10.1371/journal.pone.0221252 (PMC6707579; doi:10.1371/journal.pone.0221252)
Supplement: S3 Table — (DOCX) [file pone.0221252.s003.docx]

**S3 Table. Overview of 72 loci associated with educational attainment (Okbay et al, 2016) included into genetic risk score (GRS_Edu_).**

| **Chr:Position*** | **Original SNP** | **Proxy SNP^1^** | **Linkage disequilibrium (LD)^2^** |
| --- | --- | --- | --- |
| 1:8490603 | rs301800 |  |  |
| 1:43982527 | rs11210860 |  |  |
| 1:72733610 | rs34305371 |  |  |
| 1:72762169 | rs2568955 |  |  |
| 1:91189731 | rs1008078 |  |  |
| 1:204587047 | rs11588857 |  |  |
| 1:211613114 | rs1777827 |  |  |
| 1:243503764 | rs2992632 |  |  |
| 2:10977585 | rs76076331 |  |  |
| 2:15621917 | rs11689269 |  |  |
| 2:51873599 | rs1606974 | rs13010288 | 1.000 |
| 2:57387094 | rs11690172 |  |  |
| 2:60757419 | rs2457660 |  |  |
| 2:60976384 | rs114598875 |  |  |
| 2:61482261 | rs10496091 |  |  |
| 2:100333377 | rs13402908 |  |  |
| 2:100753490 | rs4851251 |  |  |
| 2:100821548 | rs12987662 | rs2871344 | 0.966 |
| 2:144152539 | rs17824247 |  |  |
| 2:161920884 | rs16845580 |  |  |
| 2:162818621 | rs4500960 |  |  |
| 2:193731929 | rs6739979 |  |  |
| 2:194296294 | rs2245901 |  |  |
| 2:237056854 | rs55830725 |  |  |
| 3:48623124 | rs35761247 |  |  |
| 3:48939052 | rs62259535 |  |  |
| 3:49406708 | rs148734725 |  |  |
| 3:49914397 | rs11712056 |  |  |
| 3:50075494 | rs112634398 |  |  |
| 3:85674790 | rs62263923 |  |  |
| 3:160847801 | rs6799130 |  |  |
| 4:3249828 | rs12646808 |  |  |
| 4:18037231 | rs2610986 |  |  |
| 4:28801221 | rs34072092 |  |  |
| 4:42649935 | rs3101246 |  |  |
| 4:140764124 | rs4863692 |  |  |
| 5:45188024 | rs4493682 |  |  |
| 5:57535206 | rs2964197 |  |  |
| 5:60111579 | rs61160187 |  |  |
| 5:87896602 | rs324886 |  |  |
| 5:87934707 | rs10061788 |  |  |
| 5:103947968 | rs2431108 |  |  |
| 5:113987898 | rs1402025 |  |  |
| 5:120102028 | rs62379838 |  |  |
| 6:98187291 | rs56231335 |  |  |
| 6:98584733 | rs9320913 |  |  |
| 6:153367613 | rs7767938 |  |  |
| 7:23402104 | rs2615691 |  |  |
| 7:39090698 | rs12531458 |  |  |
| 7:92654365 | rs12671937 |  |  |
| 7:128402782 | rs113520408 |  |  |
| 7:133302345 | rs17167170 |  |  |
| 7:135227513 | rs11768238 |  |  |
| 8:145712860 | rs12682297 |  |  |
| 9:1746016 | rs1871109 |  |  |
| 9:23358875 | rs13294439 |  |  |
| 9:88003668 | rs895606 |  |  |
| 9:124644562 | rs7854982 |  |  |
| 10:103802408 | rs11191193 |  |  |
| 10:104082688 | rs12772375 |  |  |
| 11:12748819 | rs7945718 |  |  |
| 12:14653667 | rs7955289 |  |  |
| 12:56416928 | rs2456973 |  |  |
| 12:92159557 | rs7131944 |  |  |
| 12:121279083 | rs572016 |  |  |
| 12:123767929 | rs7306755 |  |  |
| 13:58402771 | rs9537821 |  |  |
| 14:23373986 | rs1043209 |  |  |
| 14:27098611 | rs8005528 |  |  |
| 14:84913111 | rs17119973 |  |  |
| 17:43991515 | rs192818565 |  |  |
| 18:35186122 | rs12969294 |  |  |
| 21:42620520 | rs2837992 |  |  |
| 22:29880773 | rs165633 |  |  |

*Position of build 36, ^1^Proxy of original SNP, used when original SNP was not genotyped within study population, ^2^Linkage disequilibrium between original and proxy SNP according to 1000 Genome, Phase 3
