# Supplementary material for: A genetic sum score of risk alleles associated with body mass index interacts with socioeconomic position in the Heinz Nixdorf Recall Study
Source: PLoS One. 2019 Aug 23;14(8):e0221252. doi: 10.1371/journal.pone.0221252 (PMC6707579; doi:10.1371/journal.pone.0221252)
Supplement: S4 Table — (DOCX) [file pone.0221252.s004.docx]

**S4 Table.** **Sex- and age-adjusted effects and corresponding 95% confidence intervals (CI) of the genetic effect on body mass index (BMI) in linear regression models, stratified by tertiles of the BMI-associated genetic risk score (GRS_BMI_), separately for income (per 1000€) and education (per year of education).**

| **GRS_BMI_** | **n** | **ß_GRS BMI_ (95% CI)** | ***p*** |
| --- | --- | --- | --- |
| **Income** | | | |
| Low GRS_BMI_ | 1402 | -0.28 (-0.59; 0.03) | 0.08 |
| Middle GRS_BMI_ | 1422 | -0.68 (-1.02; -0.34) | 9.41*10^-5^ |
| High GRS_BMI_ | 1390 | -0.83 (-1.20; -0.47) | 9.50*10^-6^ |
| **Education** | | | |
| Low GRS_BMI_ | 1592 | -0.11 (-0.20; -0.01) | 0.02 |
| Middle GRS_BMI_ | 1520 | -0.30 (-0.40; -0.19) | 1.87*10^-8^ |
| High GRS_BMI_ | 1470 | -0.34 (-0.45; -0.23) | 2.51*10^-9^ |
