# Supplementary material for: A genetic sum score of risk alleles associated with body mass index interacts with socioeconomic position in the Heinz Nixdorf Recall Study
Source: PLoS One. 2019 Aug 23;14(8):e0221252. doi: 10.1371/journal.pone.0221252 (PMC6707579; doi:10.1371/journal.pone.0221252)
Supplement: S5 Table — (DOCX) [file pone.0221252.s005.docx]

**S5 Table.** **Sex- and age- adjusted effects and corresponding 95% confidence intervals (95% CI) on body mass index (BMI) in linear regression models including main effects and respective interaction terms of a BMI-associated genetic risk score (GRS_BMI_) and socioeconomic position-related health behaviors (no physical inactivity [PA], current smoking [S], alcohol consumption [PA; per 100g/week]).**

|  | **Model 11 -** BMI ~ GRS_BMI_+ PA + GRS_BMI_*PA + age + sex | | **Model 12 -** BMI ~ GRS_BMI_+ S + GRS_BMI_*S + age + sex | | **Model 13 -** BMI ~ GRS_BMI_+ A + GRS_BMI_*A + age + sex | |
| --- | --- | --- | --- | --- | --- | --- |
|  | **β (95%-CI)** | ***p*** | **β (95%-CI)** | ***p*** | **β (95%-CI)** | ***p*** |
| **n** | 4493 | | 4488 | | 4384 | |
| Intercept | 17.68 (14.75; 20.60) | <2.0*10^-16^ | 16.93 (14.42; 19.44) | <2.0*10^-16^ | 14.14 (11.62; 16.66) | <2.0*10^-16^ |
| GRS_BMI_ | 0.07 (0.04; 0.10) | 7.0*10^-6^ | 0.09 (0.06; 0.11) | 1.6*10-12 | 0.11 (0.08; 0.13) | <2.0*10^-16^ |
| PA | -4.17 (-8.11; -0.24) | 0.04 | - | - | - | - |
| S | - | - | -3.51 (-8.19; 1.17) | 0.14 | - | - |
| A | - | - | - | - | 0.71 (-0.02; 3.43) | 0.05 |
| GRS_BMI_ x PA | 0.06 (0.01; 0.10) | 0.01 | - | - | - | - |
| GRS_BMI_ x S | - | - | 0.03 (-0.02; 0.08) | 0.28 | - | - |
| GRS_BMI_ x A | - | - | - | - | -0.02 (-0.04; -0.001) | 0.04 |
| Age | 0.06 (0.05; 0.08) | 4.0*10^-13^ | 0.06 (0.04; 0.07) | 5.2*10-10 | 0.07 (0.05; 0.09) | 1.8*10-15 |
| Sex | -0.46 (-0.73; -0.19) | 0.001 | -0.53 (-0.80; -0.26) | 0.0001 | -0.57 (-0.86; -0.28) | 0.0001 |
