# Supplementary figures and images for: A genetic sum score of risk alleles associated with body mass index interacts with socioeconomic position in the Heinz Nixdorf Recall Study
Source: PLoS One. 2019 Aug 23;14(8):e0221252. doi: 10.1371/journal.pone.0221252 (PMC6707579; doi:10.1371/journal.pone.0221252)

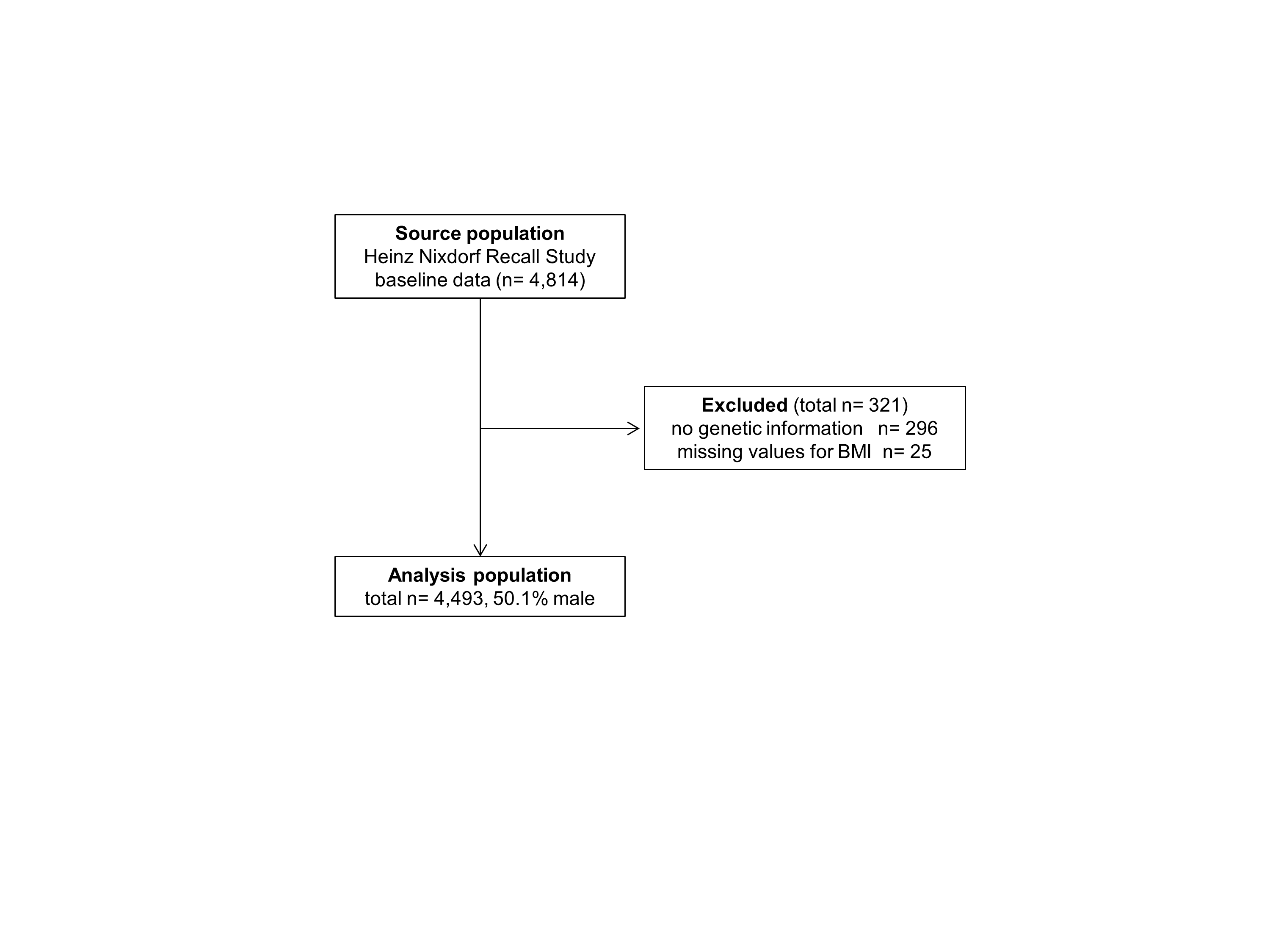

Supplement: S1 Fig — (TIF) [file pone.0221252.s006.tif]

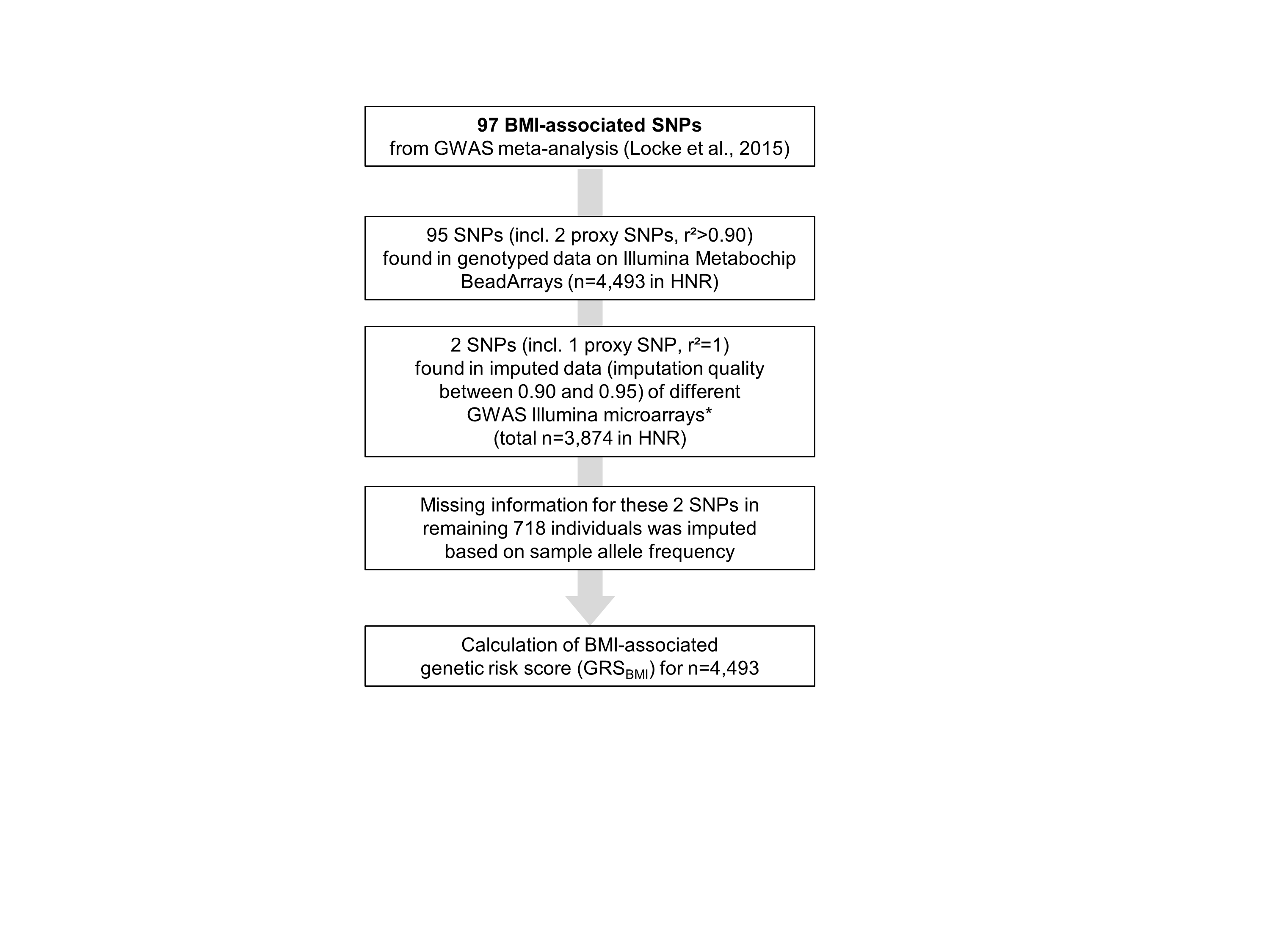

Supplement: S2 Fig — Using the meta-analyses of genome-wide association studies by Locke et al., 2015 (6), 97 SNPs representing independent genetic loci robustly associated with BMI were identified. Of these, 95 SNPs (incl. 2 SNPs were proxy SNPs [r2 > 0.9]) were found in genotyped data of the Metabochip available for all study participants included in the analysis. Two SNPs (incl. 1 SNP was a proxy SNP [r2 = 1]) were found in imputed and combined data of different Illumina genome-wide chips (Omni1-Quad n = 779, Omni1S n = 1,348, OmniExpressv1.0 n = 457, HumanCoreExome n = 1,747 with partly overlapping information available for 3,874 participants of the study population) with an imputation quality of 0.98 for both SNPs. As for these two SNPs information for 718 individuals was not available in the study population, this information was imputed based on the sample allele frequency according to the PLINK scoring routine (Purcell et al., 2007). The GRSBMI was then calculated for n = 4,493. (TIF) [file pone.0221252.s007.tif]

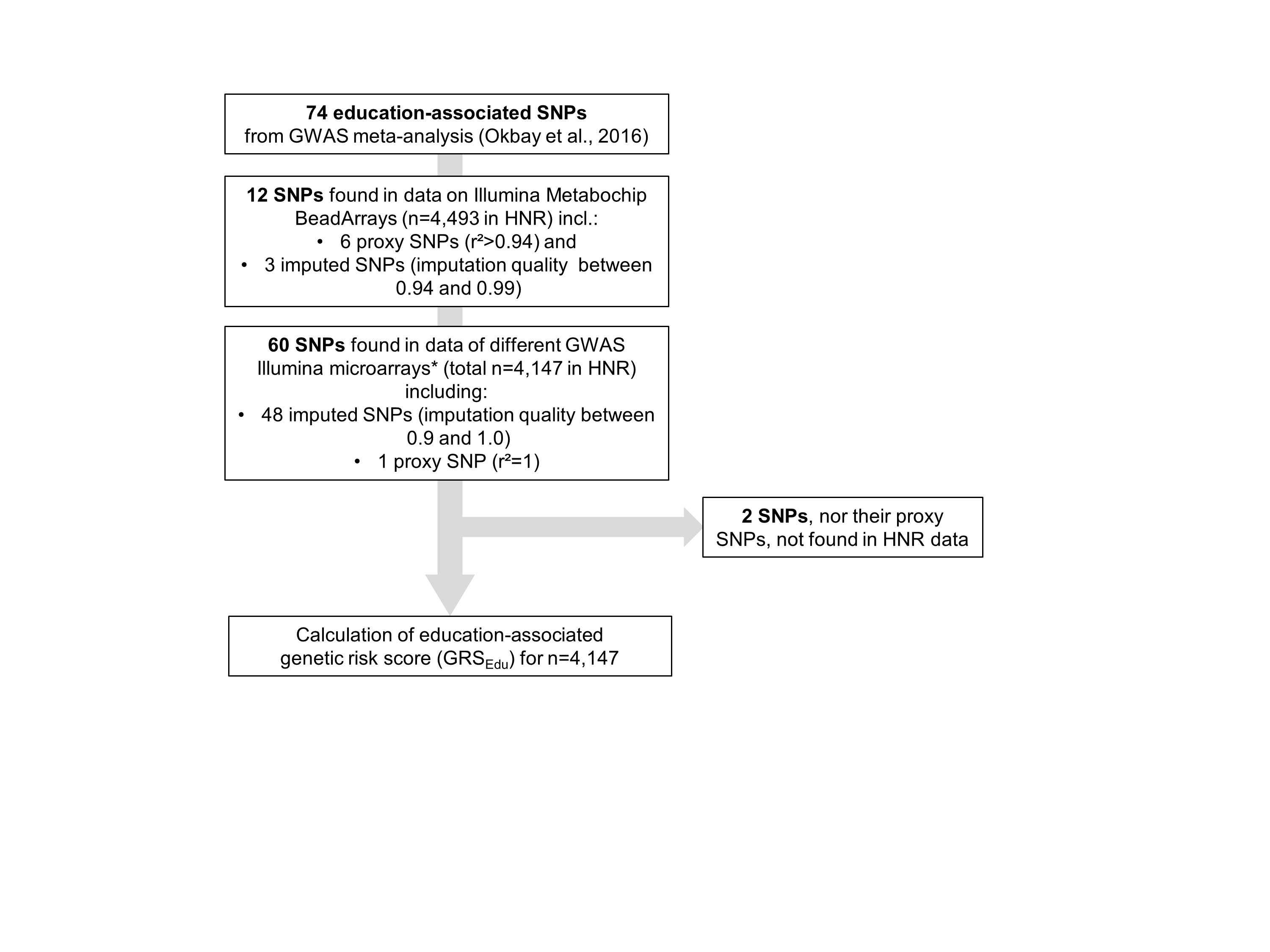

Supplement: S3 Fig — Using the meta-analyses of genome-wide association studies by Okbay et al., 2016 (24), 74 independent genetic loci robustly associated with educational attainment were selected. Of these, 12 SNPs were found in data of the Metabochip (incl. proxies for 6 SNPs [r2 between 0.94 and 1.00] and 3 imputed SNPs with an imputation quality between 0.94 and 0.99). Further, 60 SNPs were found in combined data of different Illumina genome-wide chips (Omni1-Quad n = 779, Omni1S n = 1,348, OmniExpressv1.0 n = 457, HumanCoreExome n = 1,747 with partly overlapping information available for 4,147 participants of the study population). Of these, 12 SNPs were selected from genotyped data and 48 SNPs (incl. 1 proxy SNP with r2 = 1.0) were imputed with an imputation quality between 0.92 and 1.00. For 2 loci, neither the original SNP nor the proxy SNP was available. Thus, the GRSEdu was calculated including 72 SNPs associated with educational attainment for n = 4,147. (TIF) [file pone.0221252.s008.tif]
